# Comparative evaluation of the antibacterial activity and toxicity of a large panel of aminoglycoside antibiotics

**DOI:** 10.3389/fphar.2026.1930759

**Published:** 2026-10-15

**Authors:** Nadia García-Mateo, Iris López-Hernández, Alen Čusak, Marta Fernández-Martínez, Martin Šala, Thomas Schimmang, Gregor Kosec, Štefan Fujs, Luis Martínez-Martínez, Hrvoje Petković, M. Beatriz Durán-Alonso

**Affiliations:** 1 Unit of Excellence, Institute of Biomedicine and Molecular Genetics (IBGM), University of Valladolid-CSIC, Valladolid, Spain; 2 Acies Bio, d.o.o, Ljubljana, Slovenia; 3 Service of Microbiology, University Hospital Marqués de Valdecilla, Santander, Spain; 4 Instituto de Investigacion Valdecilla (IDIVAL), Santander, Spain; 5 Department of Analytical Chemistry, National Institute of Chemistry, Ljubljana, Slovenia; 6 Unit of Microbiology, University Hospital Reina Sofía, Córdoba, Spain; 7 Instituto Maimónides de Investigación Biomédica de Córdoba (IMIBIC), Córdoba, Spain; 8 Department of Microbiology, University of Córdoba, Córdoba, Spain; 9 Biotechnical Faculty, University of Ljubljana, Ljubljana, Slovenia; 10 Department of Biochemistry and Molecular Biology and Physiology, Faculty of Medicine, University of Valladolid, Valladolid, Spain

**Keywords:** aminoglycoside, antibiotic, congener, nephrotoxicity, ototoxicity

## Abstract

**Background:**

There is a critical need for the development of novel antibiotics due to widespread occurrence of multidrug resistance. Despite their adverse effects, namely, nephrotoxicity and ototoxicity, the emergence of multi-drug resistant bacteria that are resistant to potent antibiotics such as the fluoroquinolones and the ß-lactams has highlighted the prominent role of aminoglycoside antibiotics (AGs).

**Methods:**

We carried out a comprehensive comparative characterization of the antibacterial efficacy of a whole series of AGs and AG congeners against a large panel of 61 well characterized ESKAPE clinical isolates, and investigated their ototoxic and nephrotoxic potential, using the OC-k3 and HEI-OC1 otic cell lines and the kidney LLC-PK1 cell line, respectively, all under a common set of experimental conditions; additionally, a selection of these AGs was tested on murine cochlear explants; this information is not available in the literature.

**Results:**

Out of 26 AGs tested, eight commercial AGs and four AG congeners demonstrated strong antibacterial activity against multi-drug resistant pathogens. Eight commercial AGs (amikacin, apramycin, gentamicin, isepamicin, neomycin, paromomycin, sisomicin and tobramycin) and four AG congeners (gentamicin C1a, C2, C2a and neomycin B) demonstrated strong antibacterial activity. Amikacin and isepamicin did not exert toxicity on murine cochlear explants. In addition, amikacin was the only one that did not exert significant nephrotoxicity on the LLC-PK1 cell line. Work on cochlear explants identified neomycin B and gentamicin C1a congeners as less ototoxic than their corresponding AG mixtures, although the congeners were more nephrotoxic. Importantly, we have for the first time directly compared the antibacterial activity and toxicity of seven of the most common gentamicin congeners. We identified gentamicin congeners B1 and X2 as highly toxic, while exhibiting very low antibacterial activity.

**Conclusion:**

We have demonstrated the utility of natural and semi-synthetic AGs against a large panel of multidrug-resistant clinical isolates present in our hospitals. We carried out a comparative assessment of the nephro- and ototoxicity of all these AGs and our data particularly highlighted a very high toxicity of the gentamicin congeners B1 and X2; these congeners may be present in small amounts in commercial gentamicin mixtures and their high toxicity, generally overlooked, could have serious negative implications when gentamicin formulations are applied in the clinic. Nonetheless, it must be taken into account that these studies were conducted on cell lines and, although the concentrations of AGs that are recorded in patients’ plasma are variable, -depending on factors such as dosing regimens and the infectious agent-, the AG concentrations we applied to the cultures are well above these concentrations; confirmation of these results in *in vivo* models will thus be required.

## 1 Introduction

Aminoglycoside antibiotics (AGs) bind to the bacterial 30S ribosomal subunit, resulting in RNA misreading and production of non-functional proteins, ultimately leading to bacterial cell death (Kaul et al., 2006; Kros and Steyger, 2019). A non-ribosomal effect of AGs has also been identified that conveys plasma membrane permeabilization (Zada et al., 2020). These compounds act as highly efficacious wide-spectrum antibiotics, exerting a rapid bactericidal action, as opposed to other drugs with bacteriostatic activity (Farhang et al., 2025; Huth et al., 2011; Krause et al., 2016; Serio et al., 2018). They are also hypoallergenic, which facilitates their application, and they exhibit an excellent pharmacokinetic profile and strong synergism with other antibiotics (Krause et al., 2016; Moore et al., 2025; Rivetti et al., 2023). Additionally, they convey a low production cost, which makes them first choice antibiotics in many developing countries (Adeyemo et al., 2024a; Xie et al., 2011).

Conversely, the great potential of AGs as antibiotics has been severely curtailed by some adverse effects, namely, significant nephrotoxicity and ototoxicity (Campbell et al., 2023; Fu et al., 2021; Kros and Steyger, 2019; Laurell, 2019; Steyger, 2021). Although nephrotoxicity has been reported in 10%–25% of patients treated with AGs (Dobrek, 2023; Lopez-Novoa et al., 2011; Mahi-Birjand et al., 2020; Wargo and Edwards, 2014), it is largely reversible once treatment has ceased (Mingeot-Leclercq and Tulkens, 1999; Perazella and Rosner, 2022). On the other hand, variable incidence rates of ototoxicity have been reported, ranging from 2.4%–63% of the patients receiving AG treatment (Elson et al., 2021; Huth et al., 2015; Kros and Steyger, 2019; Steyger, 2021); this variability is due to a whole series of factors, such as variations among the target population, the treatment regimens and assessment protocols, and also to a considerably frequent delay in the appearance of the first symptoms (Adeyemo et al., 2024b; Elson et al., 2021; Harruff et al., 2021; Huth et al., 2015; Laurell, 2019). Injury to the inner ear translates into hearing loss, and/or vertigo, nausea and balance deficits; unlike the situation in kidney, damage to the inner ear is irreversible, since the main targets for AGs in this organ, the so-called hair cells (HCs), do not regenerate and are permanently lost.

Despite the risk of severe side effects, the use of AGs continues to be mandatory in various instances (Disogra, 2019; EUCAST, 2020; Moore et al., 2025; Rivetti et al., 2023; Takahashi and Igarashi, 2018). This is the case when facing very serious infections such as tuberculosis, pneumonia, some urinary tract infections and sepsis. The current rise in the emergence of multi-drug resistant (MDR) bacterial strains, resistant to other types of potent antibiotics such as the fluoroquinolones and the β-lactams (Kosai et al., 2017; Levy and Bonnie, 2004; Sugden et al., 2016; Tacconelli et al., 2018; Talbot, 2013; Thy et al., 2023) has highlighted the prominent role that AGs play against these life-threatening infections (Böttger and Crich, 2019; Krause et al., 2016; Rutter et al., 2017). In addition, AGs have been evaluated as potential treatments for nonsense mutation diseases (e.g., cystic fibrosis, Duchenne muscular dystrophy, cystinosis) due to their ability to induce translational read-through of premature stop codons (Baiazitov et al., 2020; Brasell et al., 2019; Harruff et al., 2021; Li et al., 2023; Xie et al., 2011); this type of mutations have been identified in over 4,000 genes albeit there is no available therapy yet.

Considering the importance of AGs as second-line drugs to treat otherwise intractable diseases, and also considering the lack of new antibiotics (Forge and Schacht, 2000; Levy and Bonnie, 2004; Rosenberg et al., 2020; Serio et al., 2018), different avenues are being explored that aim at either reducing the toxicity caused by the currently available AGs or at developing new antibiotics that remain potent anti-infectives while reducing their toxicity (Huth et al., 2015; Kros and Steyger, 2019; Lubriks et al., 2021; Popadynec et al., 2021). A whole series of studies have been conducted where AGs have been co-administered with other compounds expected to protect the susceptible tissues from AG-induced toxicity, such as antioxidants and free radical scavengers, anti-inflammatory agents, inhibitors of cell death pathways and HC mechanoelectrical transducer (MET) channel blockers (Chowdhury et al., 2018; Gaffney et al., 2025; Jia et al., 2022; Kenyon et al., 2021; Lee et al., 2025; Mukherjea et al., 2011; Rybak and Whitworth, 2005). However, this type of therapy faces various important limitations, such as the possible appearance of non-desired effects by the co-administered drugs, and/or their interference with the beneficial action of the applied AG (Laurell, 2019; Mukherjea et al., 2011).

The generation of new, efficacious AG compounds of lesser toxicity would thus appear to be a more promising option (Lubriks et al., 2021; Sonousi et al., 2021; Yao et al., 2020); at present, this objective would be favoured by new developments in the areas of semi (synthesis) (Takemoto et al., 2022), biosynthesis, and downstream process development, enabling the isolation of individual components from complex AG mixtures (Ban et al., 2019; Guo et al., 2014; Li et al., 2018). Some of the current clinically important AGs - e.g., gentamicin (GM) - are in fact mixtures of biosynthetically and structurally related compounds that are known as AG congeners. Interestingly, it has been observed in separate studies that differences exist among AGs and their constituent AG congeners in terms of their activity, toxicity, and other pharmacokinetic properties (Bulman et al., 2020; Ishikawa et al., 2019; O’Sullivan et al., 2020; Sandoval et al., 2006).

The aim of the work here described was to carry out a comprehensive and comparative characterization of the antibacterial and toxic potential of a whole series of AG compounds, all under a common set of experimental conditions, information that is not available in the literature. Although AG-mediated ototoxicity has been addressed by numerous studies, these have mostly focused on a variety of small subsets of AGs using different methodological approaches, which makes it difficult to compare and integrate the available published data.

Importantly, we determined antibacterial efficacy against a large panel of current and well characterised clinical isolates belonging to the ESKAPE panel, which includes carbapenemase-producing enterobacteria, multidrug-resistant (MDR) *Pseudomonas aeruginosa* and MDR *Acinetobacter baumannii* (Esposito and De Simone, 2017; Tacconelli et al., 2018), pathogens that pose the greatest threat to patients’ lives. New developments in biosynthetic engineering approaches of AG biosynthesis pathways in actinomycetes, which biosynthesize these valuable antibiotics, as well as new isolation methods, which can be scaled to the industrial settings, now offer development and isolation of new natural AG analogues (congeners) at large scale, which may provide new AG drug leads (Aboshanab et al., 2024). Therefore, we also evaluated the activity of selected congeners, some of which were isolated in the scope of this work.

## 2 Materials and methods

### 2.1 Cell cultures and viability tests

Otic cell lines OC-k3 and HEI-OC1 were cultured according to Kalinec et al. (2003) (Kalinec et al., 2003), in high-glucose Dulbecco´s modified Eagle´s medium (Gibco, Grand Island, NY, USA) supplemented with 2 mM glutamine and 10% foetal bovine serum (Gibco, Grand Island, NY, USA), in the absence of antibiotic. The cells were passaged at a density of 2 × 10^5^ cells/mL and maintained in permissive conditions (i.e. 33 °C and 10% CO_2_, with the addition of 50 U/mL interferon-γ (Sigma-Aldrich, St. Louis, MO, USA) to OC-k3 cultures). The porcine proximal tubule cell line (Sigma Aldrich, St. Louis, MO, USA) LLC-PK1 was grown at 37 °C and 5% CO_2_, in Minimal Essential Medium Alpha supplemented with 10% foetal bovine serum and passaged at a density of 10^5^ cells/mL.

When conducting MTT (Sigma Aldrich (St. Louis, MO, USA)) and ATP-based (Promega Corp, Madison, WI, USA) viability assays, cultures were plated onto 96-well flat bottom plates (100 ml of cell suspension per well) 24 h prior to AG treatment; viability measurements were carried out 48 h after AG addition, following manufacturers’ instructions. Cell viability was always compared to that of non-treated control cultures that were assigned a viability of 100%. AG concentrations of 2 and 5 mM were applied to the cultures, following optimisation of the testing conditions, as described in Ishikawa et al. (2019) (Ishikawa et al., 2019).

### 2.2 AGs and isolation of AG congeners

**Commercially acquired AGs:** Amikacin (Vinci Biochem), apramycin sulfate salt (Glentham Life Science), gentamicin sulfate (Glentham Life Science), hygromycin B (Toku-E), isepamicin sulfate (Sequoia Research Products), kanamycin sulfate (Fisher BioReagents), kasugamycin hydrochloride (Toku-E), neomycin sulfate (Glentham Life Science), paromamine sulfate (Acies Bio), paromomycin sulfate (AK Scientific. Inc.), ribostamycin sulfate (Toku-E), sisomicin sulfate (Glentham Life Science), spectinomycin sulfate (Vinci Biochem), streptomycin sulfate (AK Scientific. Inc.), tobramycin (AK Scientific. Inc.), validamycin (Toku-E).

**Gentamicin congeners:** Gentamicin B sulfate, gentamicin B1 sulfate (Acies Bio Ltd.), gentamicin C1 sulfate, gentamicin C1a sulfate, gentamicin C2 sulfate, gentamicin C2a sulfate (MicroCombiChem e.K., Germany), gentamicin X2 sulfate (Acies Bio Ltd).

**Kanamycin congeners:** Kanamycin A (AK Scientific. Inc.), kanamycin B (Glentham Life Science).

**Neomycin congeners**: Neomycin B solution (2 mM) (Sigma).

### 2.3 Isolation of gentamicin congeners

Fermentation broth fractions of the gentamicin mixture of congeners (GM), kindly provided by Sandoz (Lendava, Slovenia) were used as the starting point for the isolation of gentamicins B, B1 and X2, as described in Supporting Information and Supplementary Figures S4-S12.

### 2.4 Preparation of AGs for testing procedures

To prepare stock solutions, AGs were dissolved in distilled H_2_O at a concentration of 50 mg/mL, -except for neomycin B, which was bought as a 20 mg/mL solution from Sigma- and sterilized by filtration. AGs were applied to the cultures at a final concentration of 2 and 5 mM.

### 2.5 Clinical isolates used in the study

A collection of 61 isolates cultured from clinical samples (one isolate per patient) from the bacterial collection of the clinical microbiology laboratory of the University Hospital Marqués de Valdecilla, Santander, Spain, were evaluated (Ishikawa et al., 2019; Magiorakos et al., 2012). Organisms were identified with the Vitek-2 system (BioMérieux, France), following manufacturer’s indications and were conserved at −80 °C in tryptic soy broth with 10% glycerol until tested for this study. The following species with the indicated resistance phenotypes were studied: 15 *Escherichia coli*, of which 10 produced extended-spectrum β-lactamases (ESBL) and five were not ESBL-producers, 15 *K. pneumoniae* [10 ESBL-producers and 5 ESBL-not producers), 11 *Pseudomonas aeruginosa* (7 carbapenem-resistant and four carbapenem-susceptible), 10 *Acinetobacter baumannii* (5 carbapenem-resistant and five carbapenem-susceptible), and 10 *Staphylococcus aureus* (5 resistant to methicillin and five susceptible to methicillin). All *A. baumannii*, as well as all organisms producing ESBL or resistant to carbapenem (*P. aeruginosa*) or methicillin (*S. aureus*) were multiresistant as defined by Magiorakos et al. (2012) (Magiorakos et al., 2012). *Enterococcus* spp. were not considered in this study, as these organisms are considered intrinsically resistant to aminoglycosides. A detailed description of the bacterial strains may be found in Supporting Information (Supplementary Table S2).

### 2.6 Antimicrobial susceptibility testing

Minimum inhibitory concentrations (MICs) of AGs were determined by in-house standardized broth microdilution, following CLSI guidelines (Clinical and Laboratory Standards Institute, 2012). Antimicrobial agents were tested in the range of 64 to 0.5 mg/L. After bacterial inoculation, plates were incubated in air at 35 °C for 20 h. MICs were read visually as the lowest concentration of AG inhibiting bacterial growth. *E. coli* ATCC 25922, *P. aeruginosa* ATCC 27853 and *S. aureus* ATCC 29213 were used as control strains. MIC values, corresponding to concentrations inhibiting 50% (MIC50) and 90% (MIC90) of the 61 isolates, were calculated. The results are presented in Table 1 and Supplementary Table S3.

**TABLE 1:** Antibacterial activity of AG and AG congeners on a panel of multi-resistant pathogens.

​	No isolates with MICs (mg/L)									​	​	​
Aminoglycosides	<=0.5	1	2	4	8	16	32	64	>64	MIC50	MIC90	Range
Amikacin	4	5	16	9	8	7	4	4	4	4	32	< 0.5 - >64
Apramycin			3	13	20	15	4	3	3	8	32	2 - >64
Gentamicin sulfate	18	3	6	3	2	3	3	4	19	8	>64	< 0.5 - >64
Gentamicin B sulfate							1	10	50	>64	>64	32 - >64
Gentamicin B1 sulfate				8	7	5	3	3	35	>64	>64	4 - >64
Gentamicin C1 sulfate	2	14	6	2	4	2	6	3	22	32	>64	< 0.5 - >64
Gentamicin C1a sulfate	4	15	6	1	4	3	2	6	20	16	>64	< 0.5 - >64
Gentamicin C2 sulfate	14	8	6	1	5	3	1	3	20	8	>64	< 0.5 - >64
Gentamicin C2a sulfate	9	12	2	4	3	1	3	3	24	16	>64	< 0.5 - >64
Gentamicin X2 sulfate				1	9	6	2	1	42	>64	>64	4 - >64
Hygromycin B						4	11	12	34	>64	>64	16 - >64
Isepamicin	4	8	11	10	9	3	9		7	4	>64	< 0.5 - >64
Kanamycin	1	3	4	9	1	1	6	5	31	>64	>64	< 0.5 - >64
Kanamycin_A	2	1	6	8	2	2	4	5	31	>64	>64	< 0.5 - >64
Kanamycin_B	16		3	1	3	5	6	8	19	32	>64	< 0.5 - >64
Kasugamycin hydrochloride							1	1	59	>64	>64	32- >64
Neomycin_sulfate	15	7	9	5	4	2	4	4	11	2	>64	< 0.5 - >64
Neomycin	18	7	7	5	5	1	1	5	12	2	>64	< 0.5 - >64
Neomycin_B	20	6	7	6	4	1	1	6	10	2	>64	< 0.5 - >64
Paromamine							2	10	49	>64	>64	32 - >64
Paromomycin	4	6	11	12	2	1	2	0	23	4	>64	< 0.5 - >64
Ribostamycin sulfate	1	7	12	5	2	1	2	1	30	64	>64	< 0.5 - >64
Sisomicin sulfate	23	1	3	3	3	5	8	1	14	8	>64	< 0.5 - >64
Spectimomycin sulfate						9	2	8	42	>64	>64	16 - >64
Streptomycin			2	7	4	8	3	8	29	64	>64	2 - >64
Tobramycin	23	1		3	7	1	7	8	11	8	>64	< 0.5 - >64
Validamycin									61	>64	>64	>64

### 2.7 Organotypic cochlear cultures

The animal care, use and experimental protocols followed national and institutional guidelines and were reviewed and approved by the Institutional Care and Use Committee at the University of Valladolid. All experiments were performed according to European Union Directive 2010/63/EU for the protection of animals used for experimental and other scientific purposes.

Cochlear explants were prepared from P3 C57/BL6 mice, according to the protocol described in Chen et al., 2009; Chen et al., 2009). The tissue was placed on collagen drops and allowed to recover during 48 h, prior to the addition of 0.1 mM AG and cultured for an additional 23 h; control explant cultures were included in each experiment that were not exposed to AGs. An AG concentration of 0.1 mM was applied to this ototoxicity model, since this concentration of GM yielded around 50% outer HC (OHC) survival following a 23-h treatment, as indicated in Ishikawa et al. (2019) (Ishikawa et al., 2019). This concentration is in the range of peak plasma concentrations in patients treated with various AGs such as GM (around 0,07–0.09 mM (Perrier et al., 2025)), isepamicin (around 0,05–0.17 mM (Radwanski et al., 1997; Tod et al., 1996)) and amikacin (around 0,1–0.15 mM (Grenier et al., 1987; Jaiswal et al., 2022)). Following treatment, explants were fixed and surviving HCs were identified by staining the F-actin present in their stereociliary bundles with FITC-conjugated phalloidin (Molecular Probes Inc., Life Technologies, Eugene, OR, USA). Explants were mounted on microscope slides with Vectashield mounting medium containing diamidino-phenylindole staining (DAPI, Vector laboratories, Burlingame, CA, USA); surviving HCs were imaged using a Nikon Eclipse 90i widefield fluorescent microscope equipped with a DS-Ri1 digital CCD camera and counted by using ImageJ software (Rasband, W.S., ImageJ, US National Institutes of Health, Bethesda, Maryland, USA, http://imagej.nih.gov/ij/, 1997–2014). Confocal images were taken on a Leica DMI 6000B microscope with TCS SP5 X 15 confocal system and a WLL laser controlled by LAS AF software (Leica, Spain).

### 2.8 Statistical analysis

*In vitro* tests were each conducted in triplicate and at least three independent experiments (n = 3–8) were carried out with each compound. All data are presented as means ± SD. Students’ T-tests were used to compare two groups; one- and two-way ANOVA with Holm-Sidak *post hoc* analysis were used to compare three or more groups using the statistical software SigmaPlot v.11.0 (Systat). Values of p < 0.05 were considered statistically significant. Obtained p-values are shown in Supporting information (Supplementary Table S4).

## 3 Results

A wide panel of 26 AGs and isolated AG congeners were evaluated for their anti-bacterial activity, their ototoxicity and their nephrotoxicity; these are listed in Supplementary Table S1.

### 3.1 Antibacterial activity of AGs

The description of MDR pathogens that were used in this study is presented in Supporting Information and the results on antibacterial activity of AGs are shown in Table 1 and Supplementary Table S3. Out of the 26 AGs and AG congeners that were tested in the scope of this study, a number of compounds displayed good antibacterial activity against a selected panel of 61 MDR pathogens. Our study identified eight of these AGs as exerting strong activity against the panel of ESKAPE pathogens: apramycin (Apra), tobramycin (Tobra), amikacin (Amika), isepamicin (Isepa), GM, neomycin (Neo), sisomicin (Siso) and paromomycin (Paro). Apra and Amika were the most active aminoglycosides against the collection of bacterial strains tested, with a MIC_90_ value of 32 mg/L, in contrast to other antibiotics analysed for which MIC_90_ values were >64 mg/L. The lowest MIC_50_ value (2 mg/L) was observed for the two neomycins that were studied. Of note, the kanamycin B (Kan B) congener exhibited a slightly higher activity than the kanamycin mixture (Kan) and the kanamycin A (Kan A) congener. We can therefore conclude that there are AGs that display activity against a panel of MDR ESKAPE pathogens.

Interestingly, GM, a mixture of GM congeners, predominantly from the C-group of GM (Council of Europe, 2012), displayed a slightly stronger activity (MIC_50_ of 8 mg/L) compared to the isolated GM congeners C1, C1a, C2 and C2a, with MIC_50_ values ranging from 8 to 32 mg/L. Other GM congeners of the B and X series displayed very low antibacterial activity (Table 1).

### 3.2 *In vitro* evaluation of AG ototoxicity

In parallel to the tests to measure the antibacterial efficacy of AGs and their congeners, studies were conducted to evaluate their ototoxic potential. *In vitro* cell-based ototoxicity assays were carried out on two otic cell lines, HEI-OC1 and OC-k3, derived from the organ of Corti of P14 Immortomice (Kalinec et al., 1999). As already described in Ishikawa et al. (2019) (Ishikawa et al., 2019), in order to establish an optimal set of conditions to measure the ototoxic potential of the AGs, HEI-OC1 and OC-k3 cultures were initially treated for varying lengths of time (24, 48 and 72 h) with various concentrations (1, 2, 5 mM) of two well-known ototoxic AGs, GM and Neo (Ishikawa et al., 2019); metabolically active cells were detected by conducting MTT (3-[4,5-dimethylthiazol-2-yl]-2,5-diphenyltetrazolium bromide) tests. 5 mM GM and 5 mM Neo were toxic to both cell lines following 24 h-treatments, but no toxicity was detected for 1 and 2 mM AG concentrations at this timepoint. Application of 2 mM GM or Neo for 48 h resulted in a statistically significant loss of viability in the cultures, albeit this was significantly lower than with 5 mM-treatments. No increased toxicity was recorded following 72 h-treatment, compared to 48 h, and therefore *in vitro* ototoxicity testing was thereafter conducted by applying 48 h-treatments with 2 and 5 mM AG concentrations.

#### 3.2.1 Ototoxicity of AGs

In addition to the initial studies conducted with GM and Neo (Ishikawa et al., 2019), the ototoxicity of a selection of 14 AGs was evaluated under the same established experimental conditions (Figures 1A,B, Supplementary Table S3); among these, hygromycin B was confirmed as the most toxic AG on both cell lines (Supplementary Table S3 and Supplementary Figure S1), even when applied at a 0.2 mM concentration over a 24 h-period (69,81 ± 0.011% viability of OC-k3 cultures and 74,27 ± 0.002% viability of HEI-OC1 cultures). In a similar way to GM and Neo, kasugamycin and streptomycin exerted significant ototoxicity on both cell lines at both tested AG concentrations, in contrast to Kan, ribostamycin and Tobra that did not cause any significant loss in cell viability (Figures 1A,B and Supplementary Table S3). Treatment with Apra, Isepa, paromamine and Paro only induced ototoxicity when these AGs were administered at a 5 mM concentration (Figures 1A,B, Supplementary Table S3 (Ishikawa et al., 2019)). A significantly higher resistance of the HEI-OC1 line to treatment with 5 mM concentrations of paromamine (p < 0.001) and Tobra (p = 0.038) was recorded in comparison to the OC-k3 cell line (Figures 1A,B and Supplementary Table S3). Other differences were observed in the response of the 2 cell lines to AG treatment: while Amika and spectinomycin were only significantly toxic to HEI-OC1 cultures, the viability of this cell line was not affected by Siso treatment, which induced a significant decrease in the viability of OC-k3 cells; these differences were maintained at both 2 mM and 5 mM concentrations (Figures 1A,B and Supplementary Table S3). *In vitro* ototoxicity of 5 mM concentrations of each AG was subsequently evaluated by conducting a second type of viability test based on the quantification of ATP levels in the culture media; similar results to those from the MTT assays were obtained (Figure 1C and Supplementary Table S3).

**FIGURE 1:**
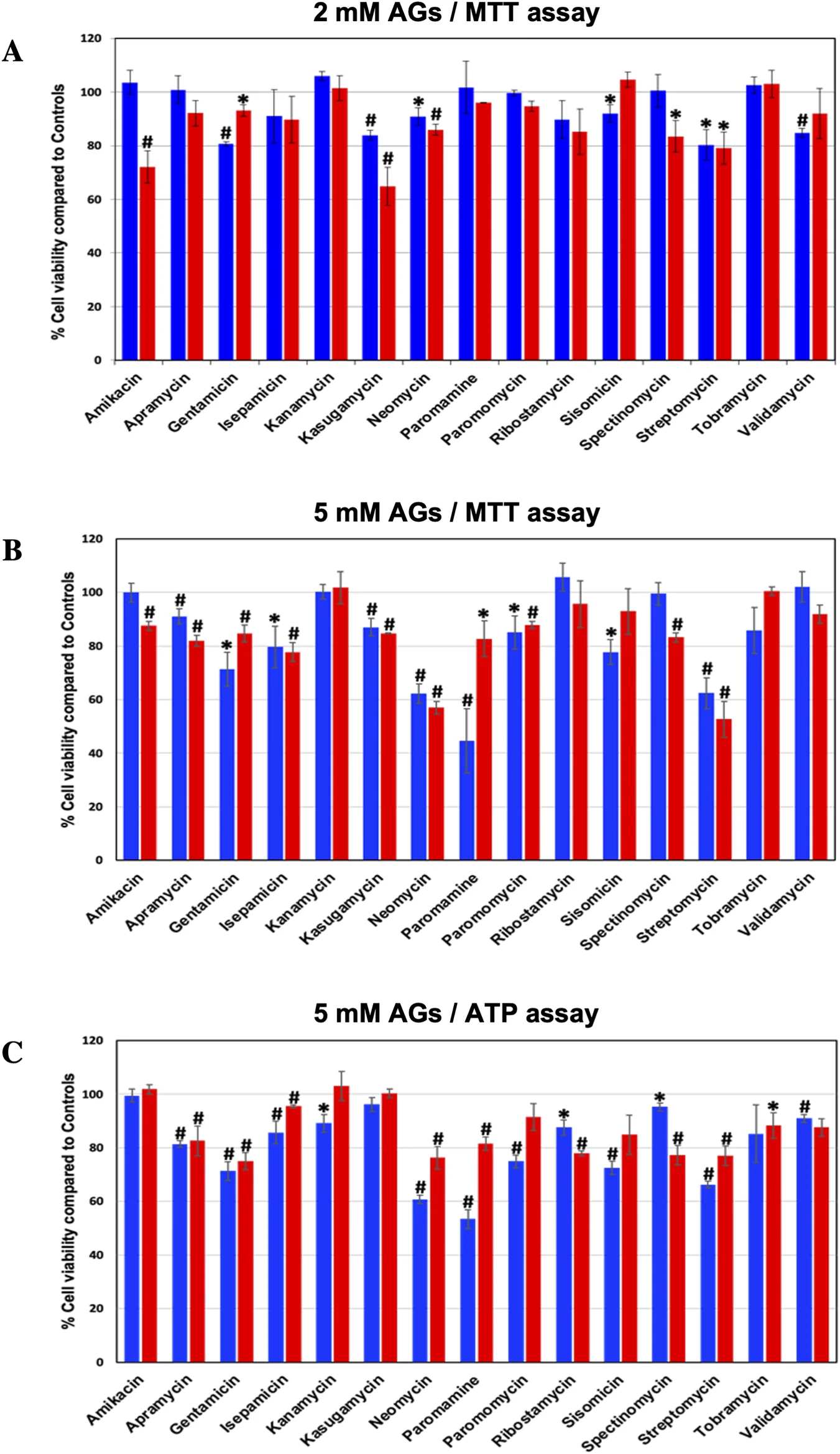
Viability of otic cell cultures following treatment with commercial AG mixtures. A series of AGs were applied at 2 mM **(A)** and 5 mM **(B,C)** concentrations to the otic cell lines OC-k3 (blue bars) and HEI-OC1 (red bars). Following 48 h-treatment, viability of the cultures was measured by conducting MTT tests **(A,B)** and by quantifying the levels of ATP present in the medium **(C)**, comparing the treated cultures to non-treated controls that were assigned a viability of 100%. Data are presented as means ± SD. *p < 0,05; #p < 0,01, as determined by two-tailed unpaired Student’s t-tests. Abbreviations: AG, aminoglycoside antibiotic; MTT, 3-[4,5-dimethylthiazol-2-yl]-2,5-diphenyltetrazolium bromide. **(A)** 2 mM concentrations/MTT assay: n = 4, except for paromamine (n = 3), Neo (n = 7), Tobra (n = 3), Paro (n = 3), streptomycin (n = 3), Kan (n = 3), Apra (n = 3). **(B)** 5 mM concentrations/MTT assay: Apra (n = 6); paromamine (n = 4 for OC-k3, n = 6 for HEI-OC1); Tobra (n = 5); kasugamycin (n = 5 for OC-k3, n = 3 for HEI-OC1); Paro (n = 5 for OC-k3, n = 4 for HEI-OC1); Isepa (n = 4 for OC-k3, n = 3 for HEI-OC1); GM (n = 3 for OC-k3, n = 5 for HEI-OC1); spectinomycin and Amika (n = 4); Kan (n = 3 for OC-k3, n = 4 for HEI-OC1); Siso, ribostamycin, validamycin, Neo and streptomycin (n = 3). **(C)** 5 mM AG concentrations/ATP assay: Kasugamycin (n = 6); Apra (n = 5); Paro (n = 4 for OC-k3, n = 6 for HEI-OC1); Tobra (n = 8 for OC-k3, n = 4 for HEI-OC1); GM (n = 4 for OC-k3, n = 3 for HEI-OC1); paromamine (n = 3 for OC-k3, n = 6 for HEI-OC1); Isepa (n = 5 for OC-k3, n = 3 for HEI-OC1); Amika, GM, spectinomycin (n = 4); Kan, Neo, ribostamycin, Siso, streptomycin, validamycin (n = 3).

We directed our attention to a series of six AGs that had shown strong antibacterial activity against the panel of ESKAPE pathogens and that had been identified by the MTT tests as (a) displaying no toxicity (Tobra), (b) being toxic to only one of the cell lines (Amika and Siso), or (c) exerting toxicity only when applied at 5 mM concentrations (Apra, Isepa and Paro) (Figures 1A,B and Supplementary Table S3). Similarly, to the results obtained with our reference AGs GM and Neo, ATP quantification indicated a significant reduction in the viability of OC-k3 and HEI-OC1 cultures treated with Apra and Isepa, as well as specific toxicity of Siso to the OC-k3 cell line; these data all agreed with the results obtained with the MTT tests (Figures 1B,C and Supplementary Table S3). On the other hand, no toxicity was recorded for Amika on any of the cell lines, in contrast to the results obtained with the MTT test following application of 2 mM and 5 mM concentrations of this AG to HEI-OC1 cultures (Figure 1 and Supplementary Table S3); neither was toxicity of Paro observed on HEI-OC1 cells when conducting the ATP-based test, as opposed to the results from the MTT assay (Figures 1B,C and Supplementary Table S3). Conversely, although no toxicity was recorded with the MTT assay when HEI-OC1 cells were treated with 5 mM Tobra and these cultures exhibited significantly higher cell viability rates than the OC-k3 cell line (p = 0.038), a significant reduction in the viability of Tobra-treated HEI-OC1 cells was recorded with the ATP-based assay. Treatment of OC-k3 cultures with 5 mM Tobra also led to a reduction in cell viability; however, this decrease did not reach statistical significance, likely due to considerably large inter-experimental variability with this AG (n = 5 for the MTT assay and n = 8 for the ATP assay) (Figures 1B,C and Supplementary Table S3).

Summarizing, we evaluated the effect of a whole series of AGs on the viability of the otic cell lines OC-k3 and HEI-OC1; MTT assays identified a series of AGs that were not toxic to any of the cell lines (Kan, ribostamycin, Tobra), toxic to both (hygromycin B, GM, Neo, kasugamycin and streptomycin), or only toxic when applied at a concentration of 5 mM (Apra, Isepa, paromamine, Paro). Interestingly, differences were recorded in the response of the two otic cell lines to some of the AGs, such as a selective toxicity of Siso to OC-k3 and not to HEI-OC1 cells; moreover, some of the differences that were observed with the HEI-OC1 cell line (i.e., with Amika, Paro and Tobra) depended on the type of viability test being conducted (Figures 1B,C and Supplementary Table S3).

#### 3.2.2 Ototoxicity of AG congeners

Significant GM-induced toxicity had been registered on both otic cell lines at 2 mM and 5 mM concentrations, as determined by the MTT and the ATP-based viability tests. The same experimental conditions that had been established for GM were used to analyse a panel of 7 GM congeners (Figure 2 and Supplementary Table S3). When tested at 2 mM AG concentrations (Figure 2A), the MTT assay identified GM B1 and GM X2 as the most toxic of all the GM congeners - (cell viabilities with GM B1 of 8,55 ± 7.66% (p = 0,003) and 26,68 ± 2,35 (p = 0.002) for OC-k3 and HEI-OC1 cultures, respectively; cell viabilities with GM X2 of 3,46 ± 2.73% (p = 0,004) and 14,30 ± 3,85 (p = 0,002), respectively). These congeners were significantly more toxic (p < 0,001) than the GM mixture that resulted in viability rates of 80,66 ± 0.84% (p = 0,004 and 93,08 ± 2.1% (p = 0,02) for the OC-k3 and the HEI-OC1 cultures, respectively. No significant toxicity was recorded for congeners GM B (cell viabilities of 87,31 ± 4.78%, p = 0.056), GM C1 (87,84 ± 7.98%, p = 0,2) nor GM C1a (93,32 ± 5.86%, p = 0,3) on OC-k3 cells (Figure 2A). Subsequent testing of GM congeners at 5 mM concentrations resulted in large reductions in cell viability (Figures 2B,C and Supplementary Table S3). Unlike most congeners that appeared to be more ototoxic than the GM mixture, GM C1a exhibited a similar toxicity to GM; this result and the strong antibacterial action demonstrated by GM C1a (Table 1 and Supplementary Table S3) constituted the basis for further studies on this congener and its identification as a strong antibacterial of lower ototoxicity than GM, as reported by our group (Ishikawa et al., 2019). Conversely, the data obtained from these studies also unveiled an extremely harmful effect of some of the minor congeners that may be present in commercial GM, such as GM B1 and X2 (Figure 2 and Supplementary Table S3).

**FIGURE 2:**
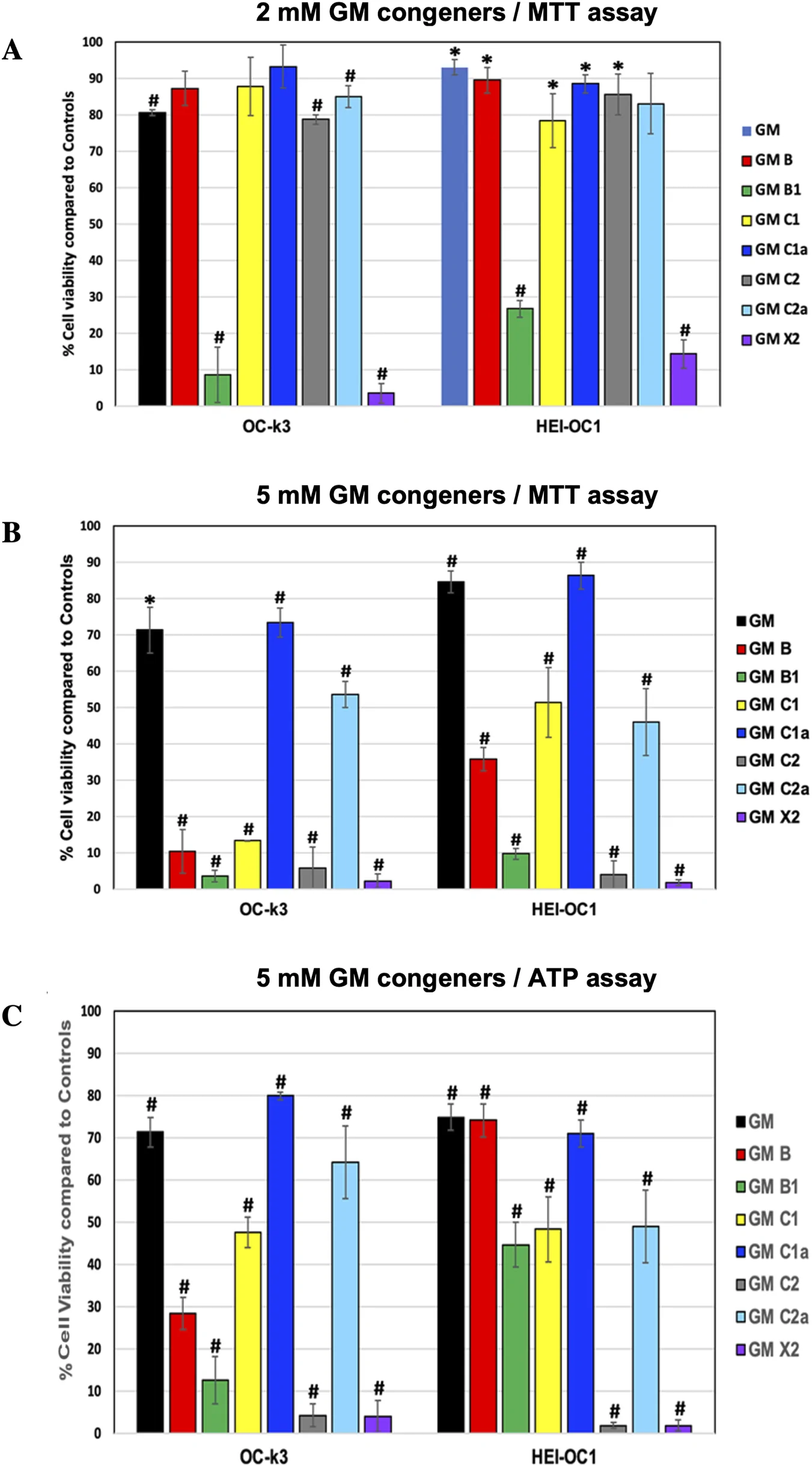
Viability of otic cell cultures following treatment with GM congeners. A series of GM congeners were applied at 2 mM **(A)** and 5 mM **(B,C)** concentrations to OC-k3 (blue bars) and HEI-OC1 (red bars) cell cultures. Following 48 h-treatment, cell viability was measured by conducting MTT tests **(A,B)** and by quantifying the levels of ATP present in the medium **(C)**, comparing the treated cultures to non-treated controls that were assigned a viability of 100%. Data are presented as means ± SD. *p < 0,05; #p < 0,01, as determined by two-tailed unpaired Student’s t-tests. Abbreviations: GM, gentamicin; MTT, 3-[4,5-dimethylthiazol-2-yl]-2,5-diphenyltetrazolium bromide. **(A)** 2 mM concentrations/MTT assay: n = 3, except for GM (n = 4), GM C1a (n = 4 for OC-k3) and GM C2 (n = 4 for HEI-OC1). **(B)** 5 mM concentrations/MTT assay: GM, GM C1a (n = 3 for OC-k3, n = 5 for HEI-OC1); GM B, GM B1, GM X2 (n = 3); GM C1 (n = 2 for OC-k3, n = 4 for HEI-OC1); GM C2, GM C2a (n = 3 for OC-k3, n = 4 for HEI-OC1). **(C)** 5 mM concentrations/ATP assay: n = 3.

In addition to these studies, the ototoxic potential of a Neo congener (Neo B) and the two Kan congeners Kan A and Kan B was tested on both the OC-k3 and the HEI-OC1 cell lines and compared to that of their corresponding parental mixtures; no significant differences were recorded (Supplementary Table S3 and Supplementary Figure 2).

Summarizing, the ototoxic potential of a series of GM congeners was evaluated. While GM C1a was identified as a very promising candidate, a small group of congeners were identified whose application led to a very significant loss of cell viability. GM B1 and X2 were significantly more toxic than any of the other AGs tested, with the exception of hygromycin B. In addition, a Neo congener, Neo B, and two Kan congeners, Kan A and Kan B, were compared to their corresponding mixtures without finding any significant differences in their ototoxicity.

### 3.3 *Ex vivo* evaluation of AG toxicity on cochlear organotypic cultures

As mentioned in Section 2.1, a series of six AGs, namely, Amika, Apra, Isepa, Paro, Siso and Tobra, were selected for further *in vitro* testing based on the results obtained from the antibacterial and cell viability studies. The ototoxic potential of these compounds was evaluated on organotypic cultures of cochlear epithelia obtained from newborn mice (postnatal day 3, P3). Besides GM C1a, which had not exerted any significant toxicity on OC-k3 cultures when applied at a 2 mM concentration, the Neo B congener was also of interest, given its strong antibacterial action and the observation that it yielded similar results to that of its parental AG.

Prior to this work, various experimental conditions were assayed, as described in Ishikawa et al. (2019) (Ishikawa et al., 2019; Lee et al., 2013; Wu et al., 2002); those that yielded outer hair cell (OHC) survival rates of around 50% following GM treatment were selected to carry out these studies. As already reported in the literature (Lee et al., 2013; Wu et al., 2002), a gradient was observed in the vulnerability of the HCs to AG toxicity, with those in the basal area of the cochlear epithelium being more susceptible to damage than the ones in the apical zone (Figure 3A).

**FIGURE 3:**
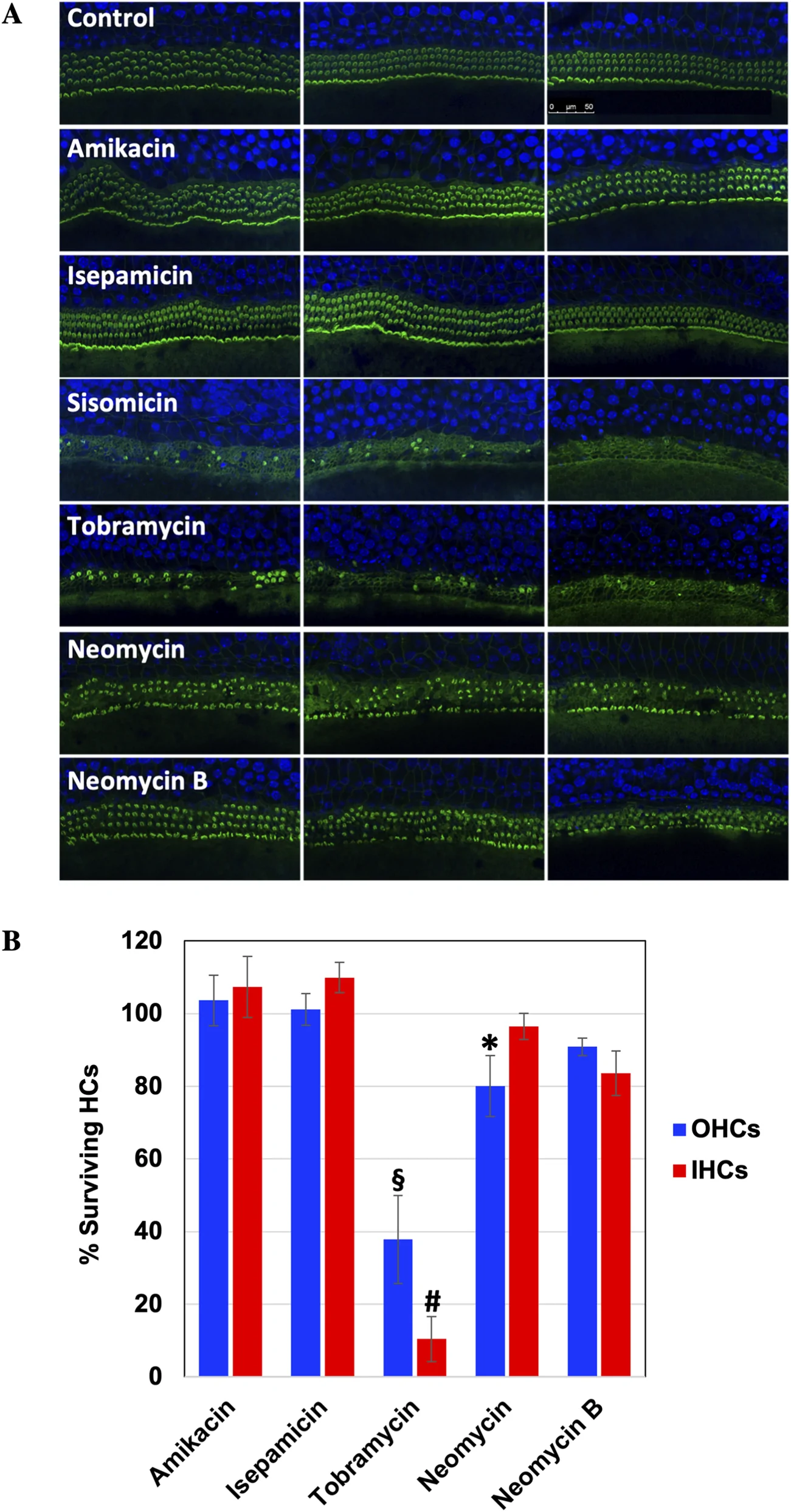
Toxicity of selected AGs on organotypic cultures of murine cochlear epithelia (n = 5 for Amika and Neo; n = 4 for Tobra; n = 3 for Isepa and Neo B). **(A)** Concentrations of 0.1 mM AGs were applied for 23 h and surviving HCs were detected following immunostaining of their stereocillia with phalloidin (from left to right: apical, middle, basal zones). Scale bar, 50 μm. **(B)** Percentages of surviving HCs in AG-treated explants were calculated relative to non-treated controls (assigned a survival of 100%). Siso-treated explants were basically devoid of any surviving HCs and therefore no counting was carried out. Data are presented as means ± SD. *p < 0,05; §p < 0,01; #p < 0.001, as determined by two-tailed unpaired Student’s t-tests.

Part of our results for Apra, Paro and GM C1a have already been published in Ishikawa et al. (2019) (Ishikawa et al., 2019), together with *in vivo* data. No toxicity was observed following treatment with Amika (% surviving OHCs = 103,62 ± 6.91%, p = 0,61; % surviving IHCs = 107,38 ± 8.42%, p = 0,41) or Isepa (% OHCs = 101,11 ± 4.37%, p = 0,81; % IHCs = 109,92 ± 4.14%, p = 0,07), while very extensive HC damage was observed when applying Tobra (% OHCs = 37,81 ± 12,07%, p = 0.002; % IHCs = 10,45 ± 6.18%, p = 0,007). As already reported for GM and GM C1a (Ishikawa et al., 2019), treatment with Tobra did not only result in reduced OHC survival but also in decreased surviving IHC rates. Treatment with Siso mostly resulted in explants that were devoid of any surviving HC and therefore counting was not carried out (Figure 3).

Interestingly, application of Neo to cultures of cochlear epithelia led to a significant loss of OHCs (OHC survival rates of 80,04 ± 8,38, p = 0,04) that was not registered following Neo B treatment (OHC survival rates of 90 ± 2,40, p = 0,06) (Figure 3).

### 3.4 *In vitro* evaluation of AG nephrotoxicity

To gain some additional information on our series of AGs and AG congeners, an initial evaluation of their nephrotoxic potential was conducted through *in vitro* viability assays on the porcine kidney cell line LLC-PK1, as described in the Materials and Methods section.

#### 3.4.1 Nephrotoxicity of AGs

MTT tests were conducted on LLC-PK1 cultures that had been treated for 48 h with 5 mM concentrations of the available AGs (Figure 4A and Supplementary Table S3). Hygromycin B proved to be the most nephrotoxic of all the AGs under study. No sign of nephrotoxicity was observed following treatment with Amika, ribostamycin, spectinomycin or validamycin; among these AGs, only the former had shown strong antibacterial activity. Significant nephrotoxicity was recorded for Kan, streptomycin and for the efficacious antibiotics Apra, GM, Isepa, Neo, Paro, Siso and Tobra. The toxicities observed following kasugamycin and paromamine treatments did not reach statistical significance, most likely due to the interexperimental variability observed with these AGs.

**FIGURE 4:**
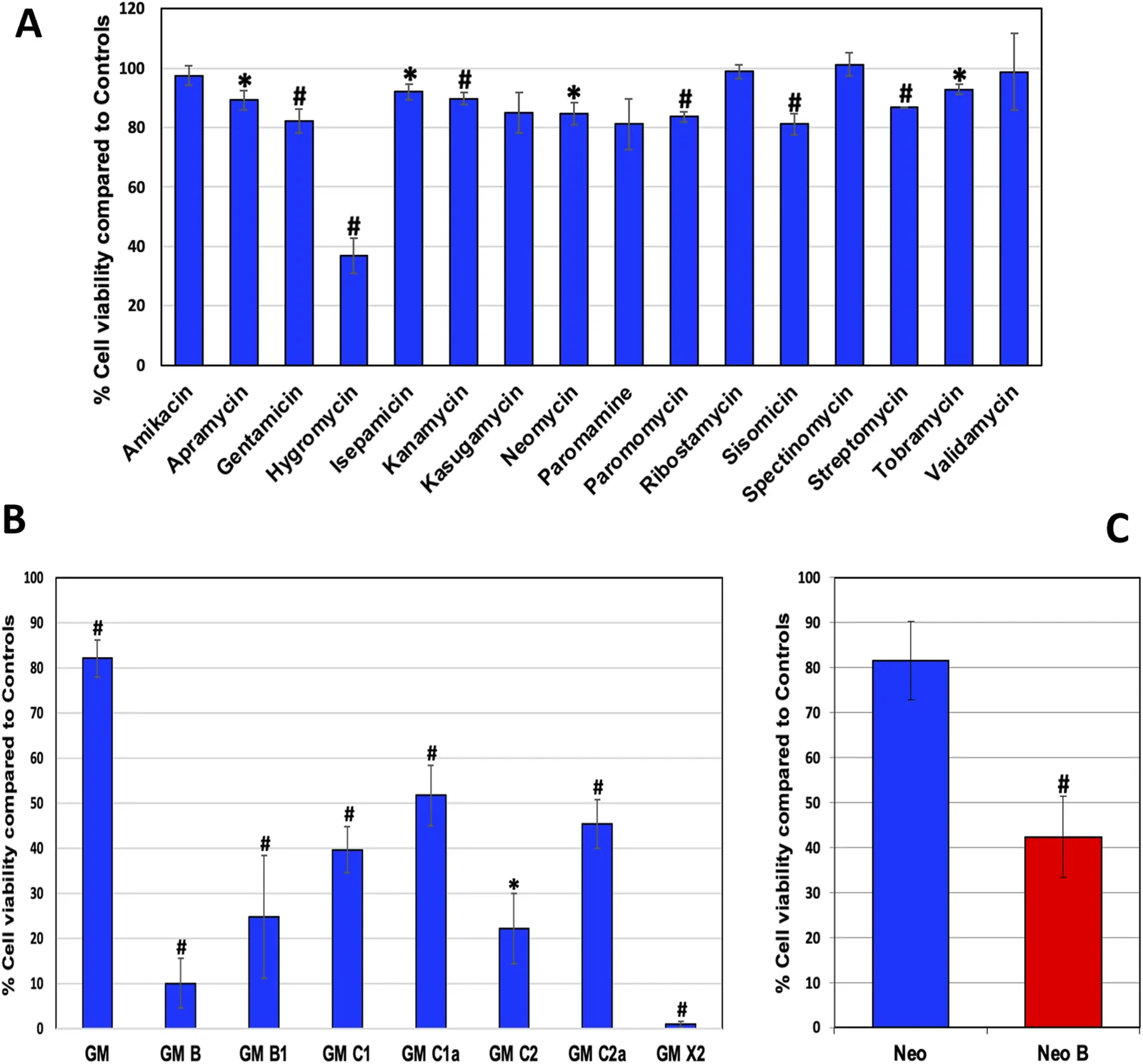
Viability of LLC-PK1 cultures following treatment with commercial AG mixtures **(A)** and with GM **(B)** and Neo **(C)** congeners. AG mixtures and GM congeners were applied at a 5 mM concentration to the kidney cell line LLC-PK1 (n = 3 except for GM, GM C1a and GM C2a, with n = 4); the neomycins in **(C)** were applied at a 2 mM concentration (n = 3). Following a 48 h-treatment, viability of the cultures was measured by using the MTT test and comparing the treated cultures to non-treated controls (viability of controls = 100%). Data are presented as means ± SD. *p < 0,05; #p < 0,01, as determined by two-tailed unpaired Student’s t-tests. Abbreviations: MTT, 3-[4,5-dimethylthiazol-2-yl]-2,5-diphenyltetrazolium bromide.

#### 3.4.2 Nephrotoxicity of AG congeners

The nephrotoxic potential of all available AG congeners was evaluated on LLC-PK1 cultures by comparison to non-treated control cultures and to cultures treated with the corresponding AG mixtures (Figures 4B,C and Supplementary Table S3). Surprisingly, treatment with a 5 mM concentration of any of the GM congeners led to a statistically significant loss in cell viability when compared to control cultures and to GM-treated cultures (Figure 4B and Supplementary Table S3). GM X2 was the most toxic, while GM C1a and GM C2a were the least toxic of the GM congeners (Figure 4B and Supplementary Table S3). GM C1a exhibited significantly lower nephrotoxicity than any other congener except GM C2a and GM C1 that demonstrated similar toxicities (p = 0,49 and p = 0,13, respectively). GM C1a was significantly less toxic than GM C2 (p = 0.001), in agreement with the observations made by Bulman and colleagues (2020) (Bulman et al., 2020) on the human renal proximal tubular cell line HK-2 and in an *in vivo* rat model. Interestingly, the commercial GM mixture displayed the lowest nephrotoxicity when compared to any of the GM congeners.

A clear loss of cell viability was observed when the Neo B congener was applied to LLC-PK1 cultures at a 2 mM concentration; this congener was significantly more toxic (p = 0,03) than the commercial Neo mixture (Figure 4C and Supplementary Table S3). Regarding the three Kan congeners tested, although no significant differences were observed among them, Kan and Kan B, unlike Kan A, yielded significant losses in cell viability compared to non-treated controls (Supplementary Figure S3 and Supplementary Table S3).

Summarizing, GM C1a and GM C2a were the least nephrotoxic of the GM congeners, although all of them were more nephrotoxic than the GM mixture. In this regard, our results differ from those reported by Bulman et al. (2020) (Bulman et al., 2020) and Sandoval et al. (2006) (Sandoval et al., 2006), who identified C2a and C2, respectively, as AGs of lower nephrotoxic potential than the GM mixture. Also, treatment of LLC-PK1 cultures with the Neo B congener resulted in a larger reduction in cell viability than that caused by the Neo mixture. No significant toxicity was recorded for the Kan A congener, in contrast to the Kan mixture and the Kan B congener.

## 4 Discussion

Despite the undesired side-effects posed by AGs (Kros and Steyger, 2019; Laurell, 2019; Steyger, 2021), these are considered antibiotics of critical importance (Serio et al., 2018; Zhao et al., 2025). Recently, plazomicin, a new semisynthetic aminoglycoside antibiotic derived from Siso by semi-synthetic modification, has been introduced in the clinic (Clark and Burgess, 2020). Etimicin is another semi-synthetic AG that is predominantly used in the clinical setting in China; it is derived from the GM C1a congener and displays better activity and a lower toxic profile (Shao et al., 2021; Zhang et al., 2025). These are good examples of how semisynthetic modification may improve the properties of natural AGs that otherwise display relatively unfavourable cytotoxic profiles (Clark and Burgess, 2020; Duscha et al., 2014; Lubriks et al., 2021; Sati et al., 2017; Zada et al., 2020) and highlight the importance of obtaining new AG drug leads.

Important advances in biosynthetic engineering of pathways involved in AG biosynthesis and downstream process development have been made in the last decades, enabling the isolation of individual AGs from complex chemical mixtures (e.g., GM) of biosynthetically and structurally related compounds known as congeners. These new approaches make it now feasible to economically isolate individual components from their corresponding AG mixtures at the industrial scale. This is especially relevant considering the observations made by various research groups that indicate that congeners from a common AG mixture may exhibit differences in their antimicrobial activity and in their potential to cause toxicity (Bulman et al., 2020; Ishikawa et al., 2019; O’Sullivan et al., 2020; Sandoval et al., 2006). The primary aim of our work was thus to evaluate a large number of AGs and their congeners, including some minor AG components.

A number of separate studies on diverse AGs have been carried out in the past; however, many of these studies lack complete and comparative information on the cytotoxicity and the antimicrobial efficacy of a wide range of AGs, most especially when referring to their activity against highly pathogenic bacteria. Within the scope of this work, we analysed a series of 26 AGs for their efficacy against a panel of 61 ESKAPE clinical isolates as well as evaluated their *in vitro* ototoxic and nephrotoxic potential; all the compounds were studied in parallel, under the same conditions. Importantly, the AG panel contained 2 Kan congeners, 1 Neo congener and 7 GM congeners; some of them have been poorly characterized to date.

We aimed to identify AGs that demonstrated a high antimicrobial activity against pathogens that render the application of this type of antibiotics mandatory, while exhibiting lower ototoxicity than currently administered AGs. Our analyses indicated that eight of the AGs exerted a strong antimicrobial action, namely, Amika, Apra, GM, Isepa, Neo, Paro, Siso and Tobra; of these, Amika was the only AG that did not demonstrate toxicity on the LLC-PK1 cell line, highlighting the medical importance of this clinically used antibiotic. In accordance with results previously published by us and other groups (Huth et al., 2011; Ishikawa et al., 2019; Kros and Steyger, 2019; Selimoglu, 2007), ATP-based cell viability tests on the otic cell lines OC-k3 and HEI-OC1 confirmed that GM and Neo were ototoxic. On the other hand, MTT assays failed to detect any ototoxicity by Tobra; although treatment with this AG resulted in decreased viability of OC-k3 cultures, Tobra was only identified as significantly toxic when using the ATP-based method to evaluate the effect of 5 mM Tobra on HEI-OC1 cultures; these results agreed with the observations made on cochlear explants. Differences between the results obtained from testing AGs on cell cultures, cochlear explants and *in vivo* models have already been reported. There are various possible explanations for these discrepancies. In the case of the organotypic cultures, they provide a more physiological setting, where the tissue´s architecture is preserved and there is a number of different cell types present; there will also be differences in the cells’ differentiation stages and in the signalling pathways that are activated in the various models, which will likely affect the response to treatment.

Similarly, to what we reported for Apra and Paro (Ishikawa et al., 2019), Isepa was toxic to both cell lines when applied at 5 mM concentrations but not when applied at the lower concentration of 2 mM; this latter result was in accordance with that obtained on organotypic cultures.

Interestingly, MTT tests identified Amika as significantly toxic only to HEI-OC1 cultures and Siso as only toxic to OC-k3 cultures. In a similar way to what had been observed with Tobra, different results were obtained on the HEI-OC1 cell line depending on the type of viability assay being conducted; the ATP-based method did not identify significant toxicity of Amika to HEI-OC1 cells, in contrast to the results obtained with the MTT assay. Taking into consideration the observations made on the cochlear explants, the ATP-based viability test would seem to yield more reliable results when studying these AGs on this cell line. Differences in the results obtained with the MTT and the ATP-based viability tests have already been reported (Petty et al., 1995; Ulukaya et al., 2008) and it has been claimed that not all the viability assays may be equally suitable at any time; this is dependent on the cell line and the cellular mechanisms that are activated by the drug under study (Chan et al., 2013; Ulukaya et al., 2008).

No discrepancy was registered between the results obtained with either viability test on the OC-k3 cell line. These and other observations such as (a) a selective toxicity of spectinomycin to HEI-OC1, AG that is reported to exhibit little ototoxicity (Au et al., 1987; Kotecha and Richardson, 1994), (b) a better correlation of the data obtained from OC-k3 cell-based assays to those recorded following treatment of cochlear explants (e.g., Amika and Siso treatments), and (c) the reported lack of significant toxicity of 2 mM GM C1a on OC-k3 cells (Ishikawa et al., 2019) and the identification of this congener as significantly less ototoxic than GM on cochlear explants and *in vivo* models (Ishikawa et al., 2019; Kobayashi et al., 2003, Kobayashi et al., 2008) would suggest that the OC-k3 cell line, although much less frequently used in ototoxicity studies than HEI-OC1 cells, may yield more predictive results than the latter.

Regarding the AG congeners, cell viability assays indicated that Neo B, similarly to what was reported for GM C1a (Ishikawa et al., 2019), possessed similar toxicities to its corresponding AG mixture. While no significant differences had been found between Neo and Neo B treatments in the cell-based assays, the application of Neo to cochlear epithelia explants led to a significant loss of OHCs that was not observed with Neo B. On the other hand, Neo B was shown to exert a strong toxicity on the kidney cell line LLC-PK1 that was significantly greater than that of the Neo mixture. All the tested GM congeners also exhibited greater nephrotoxicity than the commercial GM mixture; among them, GM C1a was the least nephrotoxic.

AG-induced nephrotoxicity is reversible, but the risk for nephrotoxicity differs between different aminoglycoside antibiotics. According to the published data, in the clinical practice, GM formulations are more nephrotoxic compared to, e.g., Tobra, Amika or netilmicin (Wargo and Edwards, 2014); these data on the nephrotoxicity of AGs generally agree with our results (see Figure 4).

It is known that AGs are taken up in the kidney into proximal tubule cells and accumulate in lysosomes, where they bind acid phospholipids, causing tubular dysfunction (Lietman and Smith, 1983). Based on this information, and considering that phospholipids are negatively charged, GM congeners with reduced cationic character should bind less strongly. However, when considering the relation of structural differences between different GM congeners and nephrotoxicity, to our knowledge, it is not possible to observe such a correlation. We found in the literature an animal study on nephrotoxicity, comparing the activity of the commercial GM mixture and the GM congeners (e.g., C2), where no significant differences were observed (Lietman and Smith, 1983). Therefore, based on the literature and on our own data, it is not possible to bring conclusions on the nephrotoxicity of GM or separate GM congeners or on how the reversibility of renal tubular cell injury differs among the various GM congeners.

Strikingly, application of AG congeners at 2 mM concentrations demonstrated that GM B1 and GM X2 were highly ototoxic; these were in fact the most toxic of all congeners on both otic cell lines and their toxicity was significantly greater than that of the GM mixture. Of note, these compounds demonstrated very weak antibacterial activity; in addition, GM X2 was the most nephrotoxic of all GMs. These results are of the utmost relevance given that AG formulations that are administered in the clinic may contain varying amounts of individual congeners (Bulman et al., 2020; Rodriquez et al., 2015; Vydrin et al., 2003); specifically, GM consists of a mixture of five congeners of the C-subtype (C1, C1a, C2, C2a, C2b) and others found in smaller quantities such as GM A, GM B, GM B1 and GM X2 (Rodriquez et al., 2015). Data have been published that indicate differences in the anti-bacterial activity and the ototoxic and nephrotoxic potential of GM C subtypes and impurities such as Siso, which may account for variations in the antimicrobial efficacy and the toxicity of different GM preparations (Bulman et al., 2020; O’Sullivan et al., 2020). Evidence has been gathered that demonstrates a greater toxicity of congener GM C2 (Bulman et al., 2020; O’Sullivan et al., 2020); on the other hand, it has been reported that serious side effects due to GM treatment might in fact be associated to the presence of some of the minor components (O’Sullivan et al., 2020; Wienen and Holzgrabe, 2003). Our present work points to GM C2 being a very toxic component of commercial GM and goes beyond the study of GM congeners of the C-subtype, unveiling GM B1 and GM X2 as potentially the most toxic congeners in the GM mixture. The results for GM B1 and GM X2 were consistent throughout all the tests we conducted and our *in vitro* methodology was already validated when leading to the identification of GM C1a as a potentially less ototoxic AG congener than the others in our panel, results that were later on confirmed in *in vivo* studies (Ishikawa et al., 2019). Nonetheless, it must be taken into account that these studies were conducted on cell lines and, although the concentrations of AGs that are recorded in patients’ plasma are variable, -depending on factors such as dosing regimes, and the infectious agent-, the AG concentrations we applied to the cultures are well above these concentrations. Also, not all AGs demonstrate the same tissue distribution pattern and pharmacokinetics and therefore an equal concentration of AGs applied to an *in vitro* model may not necessarily correlate to the effect of those AGs on target tissues like the inner ear and the kidney. Therefore, the clinical relevance of our findings will require support from *in vivo* work (e.g., using a guinea pig model, as described in Ishikawa et al., 2019).

GM B and GM B1 are 4,6-disubstituted deoxystreptamine aminoglycosides that occur as low-level components in fermentation broths and pharmacopeial material and are controlled as impurities (Jana et al., 2023b). Recent total-synthesis work has characterized GM B1, GM C-subtypes and GM X2 for antibacterial and mitochondrial/cytoplasmic ribosomal selectivity, and the results for the GM C-subtypes have been correlated with published data on cochlear toxicity; however, no correlation has been established for undesired impurities GM B, GM B1 or GM X2 (Jana et al., 2023). Thus, to our knowledge, our data demonstrate for the first time the very high toxicity and low anti-microbial activity of GM B1 and GM X2 and compare these congeners to GM C-components.

As discussed by Jana et al. (2023a), two hypotheses have been advanced for the ototoxicity of AGs. The first one suggests that ototoxicity arises from the inhibition of protein synthesis in the cochlea by the AGs, and the second is that AG-induced ototoxicity arises through blockage of the MET channels on entry into the cochlea.

All GM congeners belong to the group of so-called 4,6 – disubstituted deoxystreptamine aminoglycosides, and so do GM B1 and GM X2. These two congeners differ from the structure of the GM C group in carbon two and carbon three of the first ring, which is attached by carbon four to carbon 6 of deoxystreptamine (second ring), analogously to Siso, which also displays very high ototoxicity. So, this is a main structural difference when comparing the structure-activity relation of GM B1, GM X2 and Siso to the large sub-group of GM C congeners, which constitute the main component of the commercial GM preparation that is used clinically. Interestingly, in the study by (Jana et al., 2023b), GM B1, which contains the 6′-amino group at a different position than GM X2, displays lower inhibitory activity against the human cytoplasmic ribosome than GM X2. In contrast, the GM X2 congener is one of the weakest inhibitors of the bacterial ribosome, i.e., it displays very low antibacterial activity. As mentioned earlier, GM X2 potentially displays higher activity against the human cytoplasmic ribosome, compared to GM B1, which could be the reason for its higher read-through activity. To conclude, the structural basis that modulates target interactions of AGs such as GM B1 and GM X2 likely depends on exact ring 1 arrangements, which likely underlines much of the toxicity ranking (Jana et al., 2023b; O’Sullivan et al., 2020). However, based on current results, and literature data, it is not possible to propose preferred mechanisms responsible for the high toxicity of these 2 GM congeners. The high toxicity of GM B1 and particularly of GM X2 to both HCs and kidney cells is likely not caused by only one single mechanism.

It is worth noting that, at present, regulatory organisations such as EMA demand the identification of GM B1 as a “specified impurity” in the GM mixture, while GM X2 is not considered (Council of Europe, 2012). Considering that the concentration of such constituents often varies in commercial GM mixtures during the industrial fermentation and downstream process, our observations indicate that even a very low content of these congeners might have a significant influence on the overall toxicity of different commercial GM preparations.

## 5 Conclusion

We have carried out the study of a whole panel of AGs and AG congeners, evaluating their antimicrobial efficacy against a collection of clinical isolates from the ESKAPE group, as well as their *in vitro* ototoxic and nephrotoxic potential. Eight commercial AGs (Amika, Apra, GM, Isepa, Neo, Paro, Siso and Tobra) and four AG congeners (GM C1a, GM C2, GM C2a, Neo B) demonstrated strong antibacterial activity. As reported for Apra and Paro (Ishikawa et al., 2019), Amika and Isepa did not exert toxicity on murine cochlear explants. In addition, Amika was the only one that did not exert significant nephrotoxicity on the LLC-PK1 cell line. Regarding our work on AG congeners, work on cochlear explants identified Neo B and GM C1a as less ototoxic than their AG mixtures, although the congeners were more nephrotoxic. Importantly, we have for the first time directly compared the antibacterial activity and toxicity of seven of the most common GM congeners. Our data on the toxicity of GM congeners GM B1 and GM X2 point to a very high toxicity of these two AGs, which has been generally overlooked and could have negative implications when GM formulations are applied in the clinic. Nonetheless, it must be taken into account that these studies were conducted in cell lines and the concentrations applied to the cultures were above those found in patients’ plasma. Given the extremely low antibacterial activity and high toxicity recorded for these minor AGs, and the important implications these results convey, *in vivo* confirmation of these results would be of the utmost relevance.

## Data Availability

The original contributions presented in the study are included in the article/Supplementary Material, further inquiries can be directed to the corresponding authors.
