# Supplementary material for: Comparative evaluation of the antibacterial activity and toxicity of a large panel of aminoglycoside antibiotics
Source: Front Pharmacol. 2026 Oct 15;17:1930759. doi: 10.3389/fphar.2026.1930759 (PMC13717151; doi:10.3389/fphar.2026.1930759)
Supplement: Supplementary file 1 [file DataSheet1.pdf]

1. Selection of aminoglycoside antibiotics (AGs) and AG congeners.

AGs
Amikacin
Apramicin Sulfate
Gentamicin Sulfate
Hygromycin B
Isepamicin Sulfate
Kanamycin Sulfate
Kasugamycin hydrochloride
Neomycin sulfate
Paromamine Sulfate
Paromomycin sulfate
Ribostamycin sulfate
Sisomicin sulfate
Spectinomycin sulfate
Streptomycin sulfate
Tobramycin
Validamycin
**GENTAMICIN CONGENERS**
Gentamicin B Sulfate
Gentamicin B1 Sulfate
Gentamicin C1 Sulfate
Gentamicin C1a Sulfate
Gentamicin C2 Sulfate
Gentamicin C2a Sulfate
Gentamicin X2 Sulfate
**KANAMYCIN CONGENERS**
Kanamycin A
Kanamycin B
**NEOMYCIN CONGENERS**
Neomycin B

Table S1. List of AGs and AG congeners that have been tested within the scope of this study.

2. Antibacterial activities of the selected aminoglycoside antibiotics against multidrug-resistant clinical isolates.

Clinical isolates used in this study

Species	**Phenotye**	**Isolate (HUM-n)**	**β-lactamase**	**AME**
E. coli	ESBL	04-2283	CTX-M-14	*ND*
E. coli	ESBL	05-238	CTX-M-14	*ND*
E. coli	ESBL	12,7	CTX-M-15	*ND*
E. coli	ESBL	16,7	CTX-M-15	*ND*
E. coli	ESBL	12,8	SHV-12	*ND*
E. coli	ESBL	06.03	CTXM-15	*aac6-Ib*
E. coli	ESBL	16.06	CTXM-15	*aac6-Ib, aac3-IIa*
E. coli	ESBL	24.11	SHV-12	*aac3-IIa, aph3-IIa*
E. coli	ESBL	32.11	CTXM-9, SHV12	*aac6-Ib, ant2"-Ia*
E. coli	ESBL	49.09	CTXM-15	*ant2"-Ia*
E. coli	AMP, FQ, SXT	14-2566	/	*ND*
E. coli	AMP, FQ, SXT	14-2567	/	*ND*
E. coli	AMP, FQ, SXT	14-3249	/	*ND*
E. coli	AMP, FQ, SXT	14-3702	/	*ND*
E. coli	AMP, FQ, SXT	14-4412	/	*ND*

K. pneumoniae	ESBL	8,14	CTX-M-15	*ND*
K. pneumoniae	ESBL	11,11	CTX-M-15	*ND*
K. pneumoniae	ESBL	12,17	CTX-M-15	*ND*
K. pneumoniae	ESBL	6,10	SHV-12	*ND*
K. pneumoniae	ESBL	11,03	SHV-12	*ND*
K. pneumoniae	ESBL	28.5	SHV-12	*aac6-Ib, ant2''-Ia, aph3-Ia*
K. pneumoniae	ESBL	25.3	CTXM-15, TEM-74	*aac6-Ib, aph3-Ia*
K. pneumoniae	ESBL	6.19	TEM-15/ SHV-1	*aac6-Ib, aac3-IIa*
K. pneumoniae	ESBL	38.24	TEM-52	*ant2''-Ia, aac3-IVa*
K. pneumoniae	ESBL	49.03	CTXM-14, SHV-12	*aph3-Ia, aac3-IVa*
K. pneumoniae	pABL (+)	282	/	*ND*
K. pneumoniae	pABL (+)	284	/	*ND*
K. pneumoniae	pABL (+)	286	/	*ND*
K. pneumoniae	pABL (+)	291	/	*ND*
K. pneumoniae	pABL (+)	293	/	*ND*
A. baumannii	Carbapenem-R	17/AGL002	/	*ND*
A. baumannii	Carbapenem-R	31/JUA002	/	*ND*

A. baumannii	Carbapenem-R	59/RAC011	/	*ND*
A. baumannii	Carbapenem-R	133/SOR005	/	*ND*
A. baumannii	Carbapenem-R	152/PUM009	/	*ND*
A. baumannii	Carbapenem-S	5/CAR005	/	*ND*
A. baumannii	Carbapenem-S	7/LAL001	/	*ND*
A. baumannii	Carbapenem-S	12/SCQ004	/	*ND*
A. baumannii	Carbapenem-S	16/AGL001	/	*ND*
A. baumannii	Carbapenem-S	19/CLI001	/	*ND*
P. aeruginosa	Carbapenem-R	12	/	*ant2"-Ia*
P. aeruginosa	Carbapenem-R	19	/	*ND*
P. aeruginosa	Carbapenem-R	36	/	*None detected*
P. aeruginosa	Carbapenem-R	60	/	*ND*
P. aeruginosa	Carbapenem-R	238	/	*aac6-Ib, aac3-IIa, ant2"-Ia*
P. aeruginosa	Carbapenem-R	81	/	*aac6-Ib*
P. aeruginosa	Carbapenem-R	88	/	*ant2"-Ia*
P. aeruginosa	Carbapenem-S	22	/	*ant2"-Ia*
P. aeruginosa	Carbapenem-S	90	/	*aac6-Ib*

P. aeruginosa	Carbapenem-S	75	/	*ND*
P. aeruginosa	Carbapenem-S	221	/	*ND*
S. aureus	*MRSA* Linezolid-S	04-219	/	*ND*
S. aureus	*MRSA* Linezolid-S	04-47	/	*ND*
S. aureus	*MRSA* Linezolid-R	07-1807	/	*ND*
S. aureus	*MRSA* Linezolid-R	09-6253	/	*ND*
S. aureus	*MRSA* Linezolid-R	09-6297	/	*ND*
S. aureus	FQ, ERY	10-6939	/	*ND*
S. aureus	FQ, ERY	11-224	/	*ND*
S. aureus	FQ, ERY	11-3395	/	*ND*
S. aureus	FQ, ERY	12-0175	/	*ND*
S. aureus	FQ, ERY	14-1123	/	*ND*

ND – not determined

Table S2. Information on the clinical isolates used in this study. Description of the 61 clinical isolates cultured from clinical samples (one isolate per patient) from the bacterial collection of the clinical microbiology laboratory at the University Hospital Marqués de Valdecilla, Santander, Spain, which were evaluated (Ishikawa et al., 2019; Magiorakos et al., 2012). Aminoglycoside modified enzymes (AME) were not investigated for all clinical isolates.

No.	AG	MIC50[a]	MIC90[a]	OTOTOXICITY (OC-K3)[b]		OTOTOXICITY (HEI-OC1)[c]		Nephrotoxicity (LLC-PK1)[d]
				MTT Test	ATP Test	MTT Test	ATP Test	MTT Test
1	Amikacin	**4**	**32**	99,93 ± 3,59	99,5 ± 2,30	87,54 ± 1,73	101,85±1,64	97,47 ± 3,24
2	Apramicin sulfate	**8**	**32**	91,01 ± 2,80	81,43 ± 1,25	81,95 ± 1,96	82,62 ± 5,59	89,21 ± 3,3
3	Gentamicin sulfate C1, C2, C2a, C1a (1:1:1:1)	**8**	**>64**	71,35 ± 6,35	71,35 ± 3,49	84,63 ± 3,07	74,91 ± 3,16	82,13 ± 4,06
4	Gentamicin B sulfate	**>64**	**>64**	10,41 ± 6,01	28,40 ±3,82	35,75 ± 3,16	74,14 ± 3,94	10,03 ± 5,45
5	Gentamicin B1 sulfate	**>64**	**>64**	3,55 ± 1,69	12,59 ± 5,61	9,70 ± 1,54	44,64 ±5,35	24,75 ± 13,64
6	Gentamicin C1 sulfate	**32**	**>64**	13,41 ± 0,02	47,57 ± 3,54	51,47 ± 9,62	48,31 ± 7,77	39,65 ± 5,07
7	Gentamicin C1a sulfate	**16**	**>64**	73,34 ± 3,98	79,94 ± 0,87	86,38 ± 3,71	71,02 ± 3,28	51,74 ± 6,74
8	Gentamicin C2 sulfate	**8**	**>64**	5,75 ± 5,74	4,24 ± 2,65	3,93 ± 3,79	1,79 ± 0,70	22,18 ± 7,88

9	Gentamicin C2a sulfate	**16**	**>64**	53,60 ± 3,55	64,22 ± 8,60	46,01 ± 9,28	48,97 ± 8,69	45,33 ± 5,43
10	Gentamicin X2 sulfate	**>64**	**>64**	2,21 ± 1,91	4,00 ±3,73	1,71 ± 0,86	1,83 ± 1,37	0,91 ± 0,69
11	Hygromycin B	**>64**	**>64**					36,81 ± 5,91
	Hygromycin B **1mM**			8,28 ± 5,45	2,72 ± 0,56	13,55 ± 0,8	41,77±14,72	
	Hygromycin B **0,5mM**			13,66 ± 1,19	25,11 ± 2,23	25,68 ± 4,47	57,34 ± 1,40	
	Hygromycin B **0,2mM**			49,24 ± 6,15	60,68 ± 3,53	59,02 ± 4,17	87,57 ± 0,77	
12	Isepamicin sulfate	**4**	**>64**	79,62 ± 7,83	85,64 ± 4,17	77,67 ± 3,51	95,49 ± 0,49	91,97 ± 2,76
13	Kanamycin sulfate	**>64**	**>64**	100,31 ± 2,71	89,15 ± 3,36	101,78 ± 5,98	103,02±5,51	89,75 ± 1,9
14	Kanamycin A	**>64**	**>64**	102,94 ± 2,13	92,93 ± 1,99	104,48 ± 2,45	120,07±4,32	91,66 ± 4,66
15	Kanamycin B	**32**	**>64**	108,81 ± 3,77	89,17 ± 3,62	104,25 ± 5,45	124,24 ± 2,47	87,94 ± 3,85
16	Kasugamycin hydrochloride	**>64**	**>64**	86,96 ± 3,32	96,17 ± 2,55	84,73 ± 0,184	100,43 ± 1,59	84,93 ± 6,72

17	Neomycin sulfate	**2**	**>64**	62,24 ± 3,61	60,77 ± 1,6	57,02 ± 2,37	76,29 ± 4,21	84,73 ± 3,67
	Neomycin sulfate **2mM**			90,89 ±3,259	90,96 ± 3,66	86,01 ± 2,067	93,78 ± 5,56	81,63 ± 8,70
18	Neomycin B solution							
	Neomycin B solution **2mM**	**2**	**>64**	90,58 ± 2,45	85,14 ± 4,9	85,91 ±2,63	93,98 ± 6,53	42,35 ± 9,01
19	Paromamine sulfate	**>64**	**>64**	44,66 ± 11,96	53,40 ± 3,50	82,67 ± 6,703	81,55 ± 2,55	81,14 ± 8,47
20	Paromomycin sulfate	**4**	**>64**	85,04 ± 6,22	74,93 ± 2,35	87,86 ± 1,37	91,46 ± 4,92	83,59 ± 1,67
21	Ribostamycin sulfate	**64**	**>64**	105,73 ± 5,23	87,55 ± 2,76	95,63 ± 8,65	78,00 ± 0,87	98,87 ± 2,29
22	Sisomicin sulfate	**8**	**>64**	77,71 ± 4,65	72,58 ± 2,45	92,94 ± 8,4	84,89 ± 7,33	81,10 ± 3,53
23	Spectinomycin sulfate	**>64**	**>64**	99,52 ± 4,17	95,24 ± 1,44	83,18 ± 1,62	77,22 ± 3,67	101,25 ± 3,92
24	Streptomycin sulfate	**64**	**>64**	62,41 ± 5,70	66,24 ± 1,25	52,66 ± 6,71	77,06 ± 3,57	86,87 ± 0,05

25	Tobramycin	**8**	**>64**	85,70 ± 8,57	85,24 ± 10,79	100,53 ± 1,53	88,33 ± 4,73	92,85 ± 1,71
26	Validamycin	**>64**	**>64**	102,11 ± 5,67	90,94 ± 1,48	91,84 ± 3,43	87,56 ± 3,2	98,71 ± 12,81

Table S3. *In vitro* biological profiling of AGs and isolated AG congeners. a) MIC values (mg/L) for multidrug-resistant pathogens; b) % Viable cells compared to untreated controls, as measured in ototoxicity tests (MTT and ATP) using the OC-K3 cell line; c) % Viable cells compared to untreated controls, as measured in ototoxicity tests (MTT and ATP) using the HEI-OC1 cell line; d) % Viable cells compared to untreated controls, as measured in nephrotoxicity tests (MTT) using the LLC-PK1 cell line. All AGs tested in b, c, d, were applied at a concentration of 5 mM, except where otherwise indicated.

3. *In vitro* evaluation of AG ototoxicity

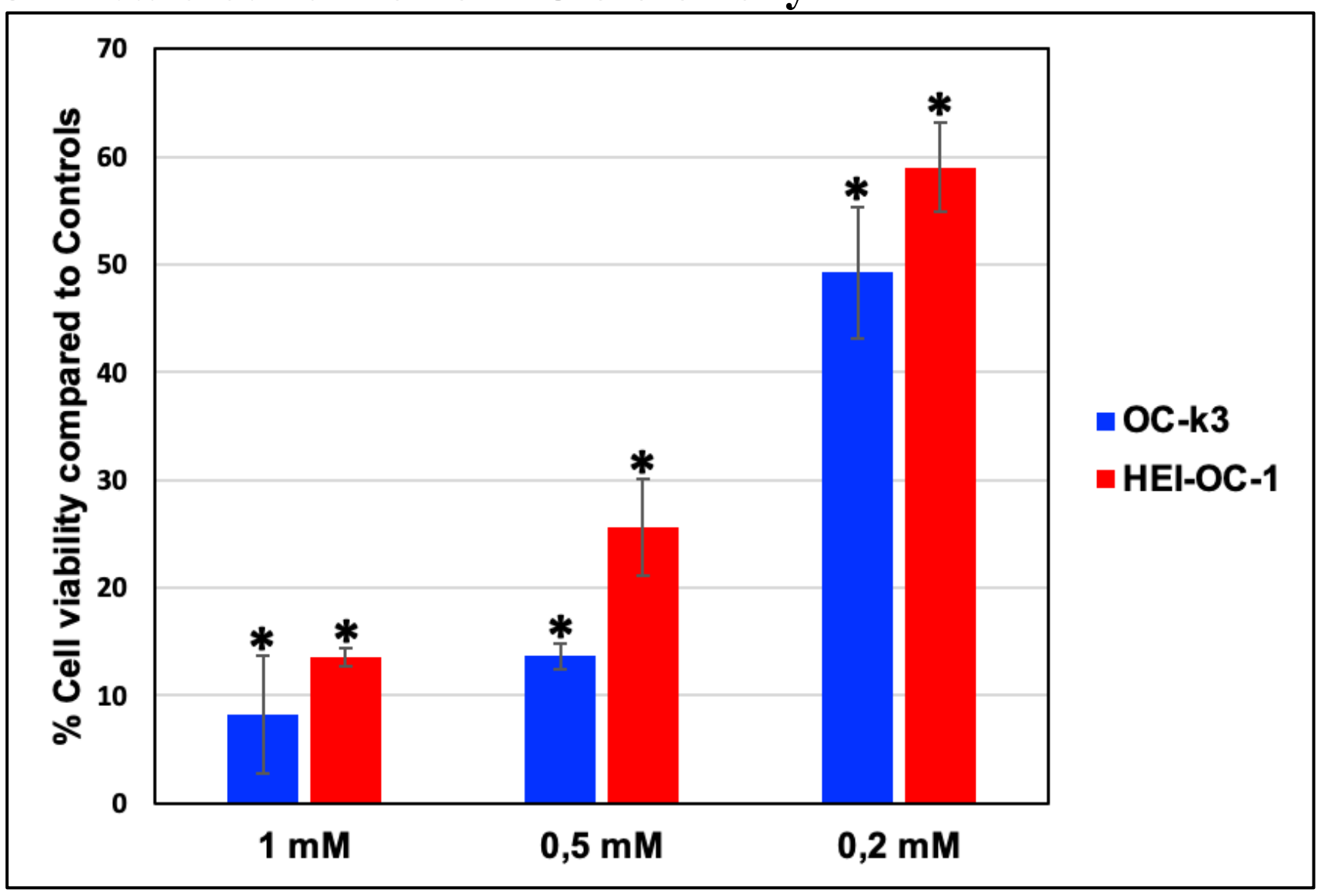

Figure S1. Viability of otic cell line cultures following treatment with hygromycin B. Various concentrations of hygromycin B were applied to OC-k3 and HEI-OC1 cultures for 48 h and cell viability was measured by conducting MTT tests, comparing the treated cultures to non-treated controls that were assigned a viability of 100%. Data are presented as means±SD; *p < 0,005 (two-tailed unpaired Student's t-tests were applied to compare each treatment to controls). Abbreviations: MTT, 3-[4,5-dimethylthiazol-2-yl]-2,5-diphenyltetrazolium bromide.

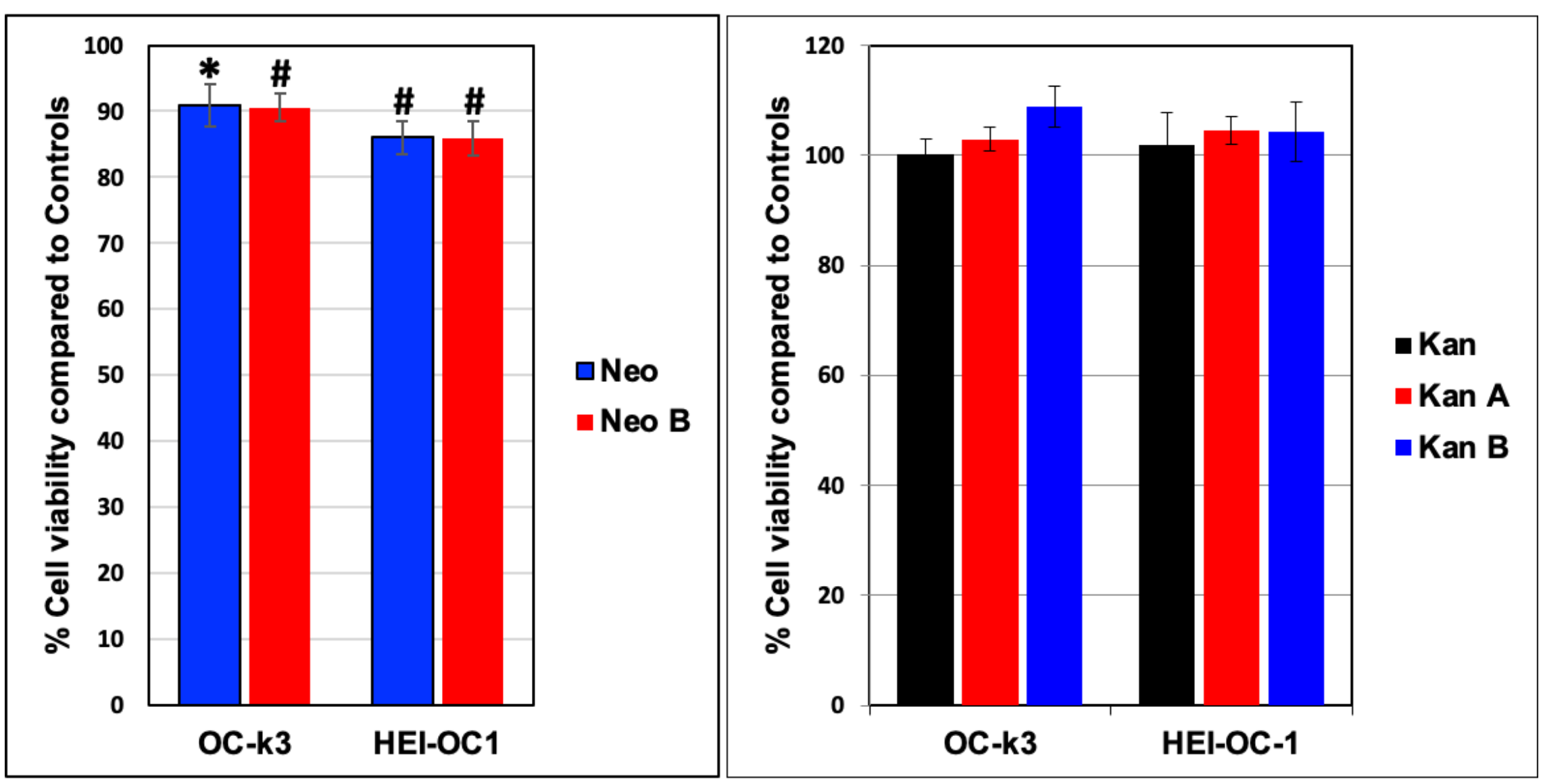

Figure S2. Viability of otic cell cultures following treatment with neomycin and kanamycin congeners. The effect of a 48 h-treatment with AG congeners Neo B, Kan A and Kan B on the viability of OC-k3 and HEI-OC1 cultures was recorded and compared to that of their corresponding parental mixtures. Neomycins were applied at a 2 mM concentration and kanamycins at a 5 mM concentration. Cell viability was measured by conducting MTT tests and comparing the treated

cultures to non-treated controls that were assigned a viability of 100%. Data are presented as means±SD; *p<0,05; #p<0,01, as determined by two-tailed unpaired Student's t-tests. Abbreviations: Neo, neomycin; Kan, kanamycin; MTT, 3-[4,5-dimethylthiazol-2-yl]-2,5-diphenyltetrazolium bromide.

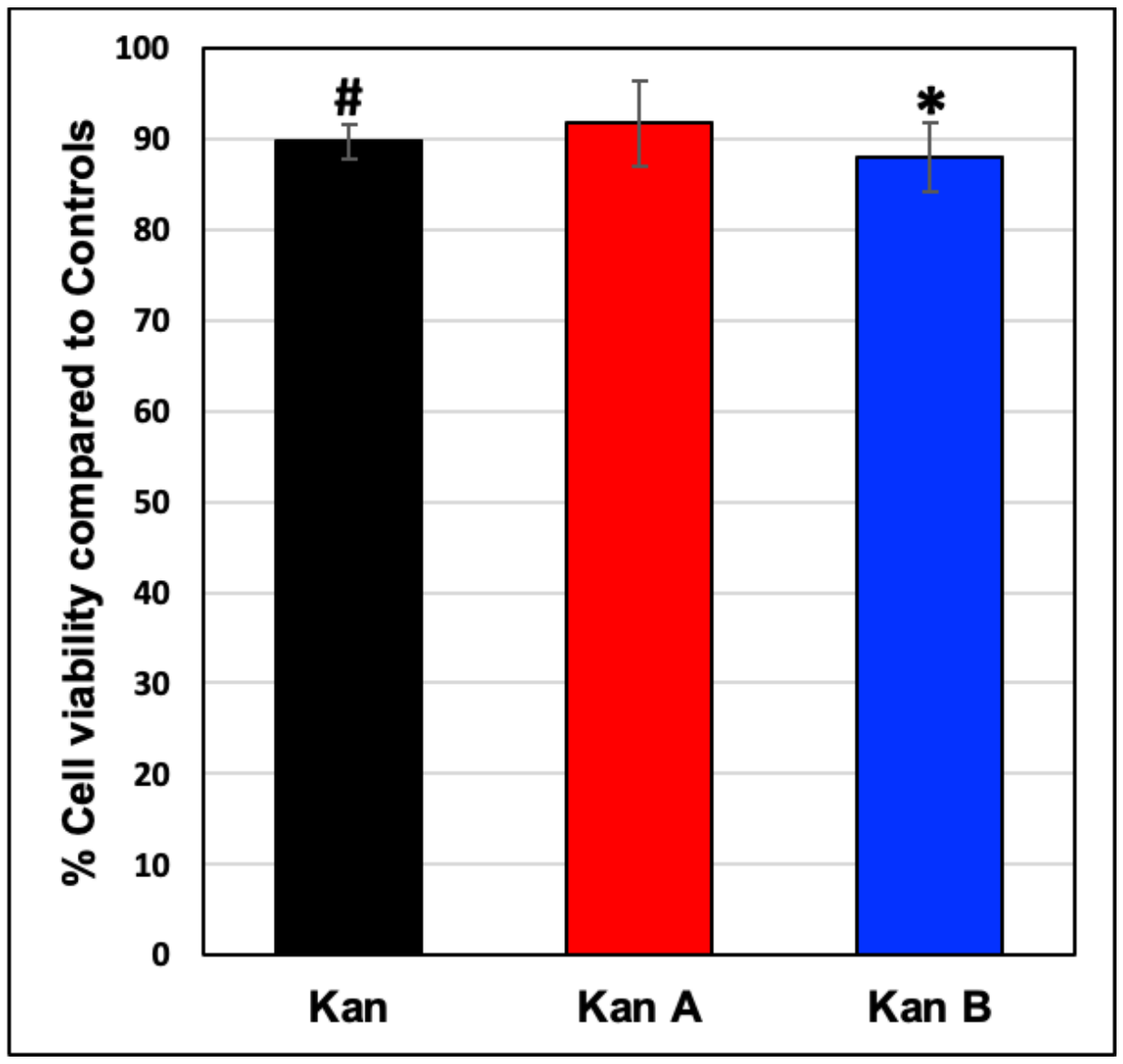

Figure S3. Viability of LLC-PK1 cultures following treatment with Kan and its congeners Kan A and Kan B. Kanamycins were applied to LLC-PK1 cultures at a concentration of 5 mM. Following a 48 h-treatment, the viability of the cultures was measured by using the MTT test and comparing treated cultures to non-treated controls that were assigned a viability of 100%. Data are presented as means±SD. *p<0,05; #p<0,01, as determined by two-tailed unpaired Student's t-tests. Abbreviations: Kan, kanamycin; MTT, 3-[4,5-dimethylthiazol-2-yl]-2,5-diphenyltetrazolium bromide.

4. Statistical results from *in vitro* and *ex vivo* toxicity studies

In vitro ototoxicity	2 mM AGs/MTT assay p values (*vs* controls)	
	OC-k3	HEI-OC1
**Amikacin**	0,386732179	0,002478289
**Apramycin**	0,847995197	0,108111861
**Gentamicin**	0,01308966	0,016417865
**Isepamicin**	0,337011706	0,28385305
**Kanamycin**	0,069552696	0,78642889
**Kasugamycin**	5,24753E-05	0,002679605
**Neomycin**	0,01619154	1,99494E-05

**Paromamine**	0,891919552	0,052283267
**Paromomycin**	0,800054583	0,051244079
**Ribostamycin**	0,148323897	0,128686141
**Sisomicin**	0,029831304	0,14150486
**Spectinomycin**	0,926528576	0,03173959
**Streptomycin**	0,008437844	0,008631395
**Tobramycin**	0,362015155	0,503675801
**Validamycin**	3,88486E-05	0,423560474
***In vitro* ototoxicity**	**5 mM AGs/MTT assay**	
	**p values (*vs* controls)**	
	**OC-k3**	**HEI-OC1**
**Amikacin**	0,98400101	0,0003677
**Apramycin**	0,009356053	3,32731E-06
**Gentamicin**	0,010723485	0,001041153
**Isepamicin**	0,040554144	0,005287824
**Kanamycin**	0,913352011	0,775769042
**Kasugamycin**	0,004375812	1,28143E-07
**Neomycin**	0,000471992	5,48018E-05
**Paromamine**	0,003589165	0,027201281
**Paromomycin**	0,042794655	0,000115263
**Ribostamycin**	0,335176842	0,640628241
**Sisomicin**	0,00867397	0,450621713
**Spectinomycin**	0,91125189	4,60E-05
**Streptomycin**	0,002738273	0,002133155
**Tobramycin**	0,133577226	0,737653329
**Validamycin**	0,728804939	0,075778361
***In vitro* ototoxicity**	**5 mM AGs/ATP assay**	
	**p values (*vs* controls)**	
	**OC-k3**	**HEI-OC1**
**Amikacin**	0,835604683	0,302348078
**Apramycin**	4,16E-07	0,014446625
**Gentamicin**	0,000726958	0,001355843
**Isepamicin**	0,008830016	0,000756595
**Kanamycin**	0,031863587	0,6400000
**Kasugamycin**	0,163916	0,810741433
**Neomycin**	1,69E-05	0,004894853
**Paromamine**	0,000183691	2,83E-05
**Paromomycin**	4,00E-05	0,11350688
**Ribostamycin**	0,010703	0,001548222
**Sisomicin**	0,000361454	0,175497855
**Spectinomycin**	0,016339393	0,000810599
**Streptomycin**	1,11596E-05	0,003027587
**Tobramycin**	0,069263911	0,032827433
**Validamycin**	0,003638741	0,060395069

In vitro ototoxicity	2 mM AG gentamicins/MTT assay			
	p values (*vs* controls)		p values (*vs* GM)	
	OC-k3	HEI-OC1	OC-k3	HEI-OC1
**GM**	4,36372E-07	0,016384639		
**GM B**	0,056893277	0,040317499	0,167573417	0,404340813
**GM B1**	0,000275902	0,001997305	9,98976E-05	4,5803E-06
**GM C1**	0,202170086	0,042502465	0,335815011	0,078111182
**GM C1a**	0,29808703	0,010548045	0,076230758	0,22834796
**GM C2**	9,75095E-05	0,041853997	0,256825239	0,257181282
**GM C2a**	0,007043974	0,111157506	0,159639836	0,233371364
**GM X2**	3,8076E-06	2,42077E-05	6,54276E-07	6,77253E-06

In vitro ototoxicity	5 mM AG gentamicins/MTT assay			
	p values (*vs* controls)		p values (*vs* GM)	
	OC-k3	HEI-OC1	OC-k3	HEI-OC1
**GM**	0,010717375	0,001041153		
**GM B**	0,000118089	3,4712E-05	0,002229689	4,62517E-05
**GM B1**	0,00031	0	0,003831455	2,12E-06
**GM C1**	6,15891E-06	0,002351501	0,005828583	0,008431167
**GM C1a**	0,002594378	0,00627	0,802933545	0,725409046
**GM C2**	0,003688527	2,50252E-07	0,005762187	6,62E-07
**GM C2a**	0,0002	0,001136194	0,07121024	0,003332504
**GM X2**	0,00038277	3,49552E-08	0,003652479	1,01E-06

In vitro ototoxicity	5 mM AG gentamicins/ATP assay			
	p values (*vs* controls)		p values (*vs* GM)	
	OC-k3	HEI-OC1	OC-k3	HEI-OC1
**GM**	0,001205654	0,001355843		
**GM B**	0,00005	0,000595031	0,001154655	0,886239374
**GM B1**	9,91447E-05	0,000492474	0,000884732	8,20E-03
**GM C1**	0,000120283	0,002650921	0,008737572	0,033792495
**GM C1a**	2,12076E-05	0,000902753	0,075643249	0,44033632
**GM C2**	3,51591E-06	1,5132E-08	0,000106494	2,26E-05
**GM C2a**	0,01415	0,004207962	0,485346295	0,0485908
**GM X2**	0,00001	2,27642E-07	0,00019179	2,90E-05

Ex vivo ototoxicity	0,1 mM AGs/Cochlear explants	
	p values (*vs* controls)	
	IHCs	OHCs
**Amikacin**	0,40653643	0,614479674

**Isepamicin**	0,074865478	0,812167718
**Tobramycin**	6,79E-06	0,002112043
**Neomycin**	0,361652114	0,04443341
**Neomycin B**	0,116331109	0,063351543

***In vitro* nephrotoxicity**	**5 mM AGs/MTT assay**
	**p values (*vs* controls)**
	**LLC-PK1**
**Amikacin**	0,479266546
**Apramycin**	0,030943646
**Gentamicin**	0,004532626
**Hygromycin**	0,000435202
**Isepamicin**	0,044000262
**Kanamycin**	0,005677362
**Kasugamycin**	0,088315418
**Neomycin**	0,014151342
**Paromamine**	0,089848646
**Paromomycin**	0,000595671
**Ribostamycin**	0,6480875
**Sisomicin**	0,005869513
**Spectinomycin**	0,766565962
**Streptomycin**	1,75596E-05
**Tobramycin**	0,014023814
**Validamycin**	0,92485928

***In vitro* nephrotoxicity**	**5 mM gentamicins/MTT assay**	
	**p values (*vs* controls)**	**p values (*vs* GM)**
	**LLC-PK1**	**LLC-PK1**
**GM**	0,004532626	
**GM B**	7,88712E-05	0,000113277
**GM B1**	0,005277	0,005665349
**GM C1**	0,000286276	0,00117481
**GM C1a**	0,000373724	0,008315627
**GM C2**	0,01008565	0,000546739
**GM C2a**	5,58791E-05	0,001619357
**GM X2**	4,92587E-05	0,000183604

***In vitro* nephrotoxicity**	**2 mM neomycins/MTT assay**	
	**p values (*vs* controls)**	**p values (*vs* Neo)**
	**LLC-PK1**	**LLC-PK1**
**Neomycin**	0,1024	
**Neomycin B**	0,003058932	0,034960907

***In vitro* ototoxicity**	**Hygromycin/MTT assay**	
	**p values (*vs* controls)**	
	**OC-k3**	**HEI-OC1**
**1 mM**	7,33045E-05	4,33503E-08
**0,5 mM**	0,000191181	0,003604889
**0,2 mM**	0,001177284	0,000601505

In vitro ototoxicity	2 mM neomycins/MTT assay			
	p values (*vs* controls)		p values (*vs* Neo)	
	OC-k3	HEI-OC1	OC-k3	HEI-OC1
Neomycin	0,016206964	1,99494E-05		
Neomycin B	0,008466163	0,005850834	0,949440548	0,9781775

In vitro ototoxicity	5 mM kanamycins/MTT assay			
	p values (*vs* controls)		p values (*vs* Kan)	
	OC-k3	HEI-OC1	OC-k3	HEI-OC1
Kanamycin	0,913340825	0,775769042		
Kanamycin A	0,217064599	0,117736613	0,474304091	0,691299722
Kanamycin B	0,05816498	0,465755269	0,116843467	0,771205568

In vitro nephrotoxicity	5 mM kanamycins/MTT assay	
	p values (*vs* controls)	p values (*vs* Kan)
	LLC-PK1	LLC-PK1
Kanamycin	0,005669362	
Kanamycin A	0,148020072	0,7232543
Kanamycin B	0,035050845	0,694900932

Table S4. p values obtained in the *in vitro* and *ex vivo* toxicity tests carried out within the scope of this work. Indicated in red are p values $p<0,05$.

5. Isolation of the gentamicin B, B1 and X2 congeners.

Protocol for the isolation of gentamicin congeners from the fermentation broth

Following fermentation, the broth was filtered and the filtrate was discarded. Gentamicin (GM) and its congeners were separated from the biomass by acid extraction with 6N H_2SO_4 at a pH=2.0 and subsequent filtration using a filter aid. The pH of the filtrate was adjusted to 7.0 with 6N NaOH solution. The neutralized solution was then passed through a cationic exchange resin column IRC-50 Amberlite. The eluate was discarded. Aminoglycosides were eluted with H_2SO_4, typically at pH=2.0. The eluate was neutralized with 6N NaOH to pH 7.0. The neutralized IRC-50 eluate was concentrated under reduced pressure to provide a dark brown crude oil containing GM and its congeners.

Decolorization, Cbz protection and chromatographic purification protocol of the IRC-50 fraction

The crude brown fraction from the IRC-50 resin was treated with 5% activated charcoal (m/m) and stirred vigorously for 1 hour. The suspension was allowed to settle down and vacuum filtered through a fitted funnel containing Celite filter aid. Clear solution was concentrated and dried in vacuum to obtain a yellow crispy solid. The crude was dissolved in CH_3CN/H_2O 1:1 and treated with Na_2CO_3 (in excess). The yellow suspension was stirred until the Na_2CO_3 dissolved completely, resulting in a homogeneous solution. Benzyl chloroformate (in excess) was added dropwise from a syringe. The reaction mixture was allowed to stir at ambient temperature for 24 hours. The mixture was partitioned between water and ethyl acetate (EtOAc). Combined organic phases were washed with brine, dried with Na_2SO_4, filtered and concentrated. Crude yellow oil was analyzed via LC-MS and purified on normal phase silica gel using a column chromatography. The mobile phase used for this separation was a dichloromethane/methanol (DCM/MeOH) mixture. The elution started with 100% DCM and ended with DCM/MeOH 80/20, with gradual increase of methanol by 5%. Each sample was also analyzed by TLC (thin layer chromatography) and LC-MS analysis. Subsequently, the samples containing particular groups of aminoglycosides were combined, concentrated and purified using preparative HPLC.

Isolation of GM congeners present in the IRC-50 resin eluate from the biosynthetic production

Downstream processing of GM congeners from the IRC-50 eluate to GM sulfate was performed as portrayed in Figure S4. The crude brown fraction from IRC-50 resin (2000 mL) was treated with activated charcoal (0,5 % (w/w); 10,0 g) and stirred vigorously for 1 hour. The suspension was

allowed to settle down and vacuum filtered through a fritted funnel containing Celite filter aid. Clear solution was concentrated and dried *in vacuo* to obtain a pale yellow crispy solid (crude material; 7,20 g). The combined crude (5,00 g) was dissolved in CH_3CN/H_2O 1:1 (v/v; 100 mL) and treated with Na_2CO_3 (excess of reagent; 9,21 g, 86,9 mmol). The yellow suspension stirred until Na_2CO_3 dissolved completely, thus forming a homogeneous solution. Benzyl chloroformate (excess of reagent; Cbz-Cl; 97 %; 9.94 g, 56.5 mmol) was added dropwise from a syringe. The reaction mixture was allowed to stir at ambient temperature for 24 hours. The mixture was partitioned between water and ethyl acetate (EtOAc). Combined organic phases were washed with water (2x), saturated solution of ammonium chloride (1x), saturated solution of sodium chloride (1x), dried over Na_2SO_4, filtered and concentrated. Crude pale-yellow oil (12,4 g) was analyzed via HPLC/MS (Figure S5) and purified on normal phase silica gel using a column chromatography. The mobile phase utilized for this separation was dichloromethane/methanol (DCM/MeOH) mixture. The elution started with 100 % DCM and ended with DCM/MeOH 80/20, with gradual increase of methanol by 5 %. Fractions were collected into the 30 mL test tubes. In addition, fractions were analyzed by TLC (thin layer chromatography) and HPLC/MS analysis. Subsequently, the samples containing particular groups of Cbz-protected aminoglycosides were combined, concentrated and further purified using a preparative HPLC.

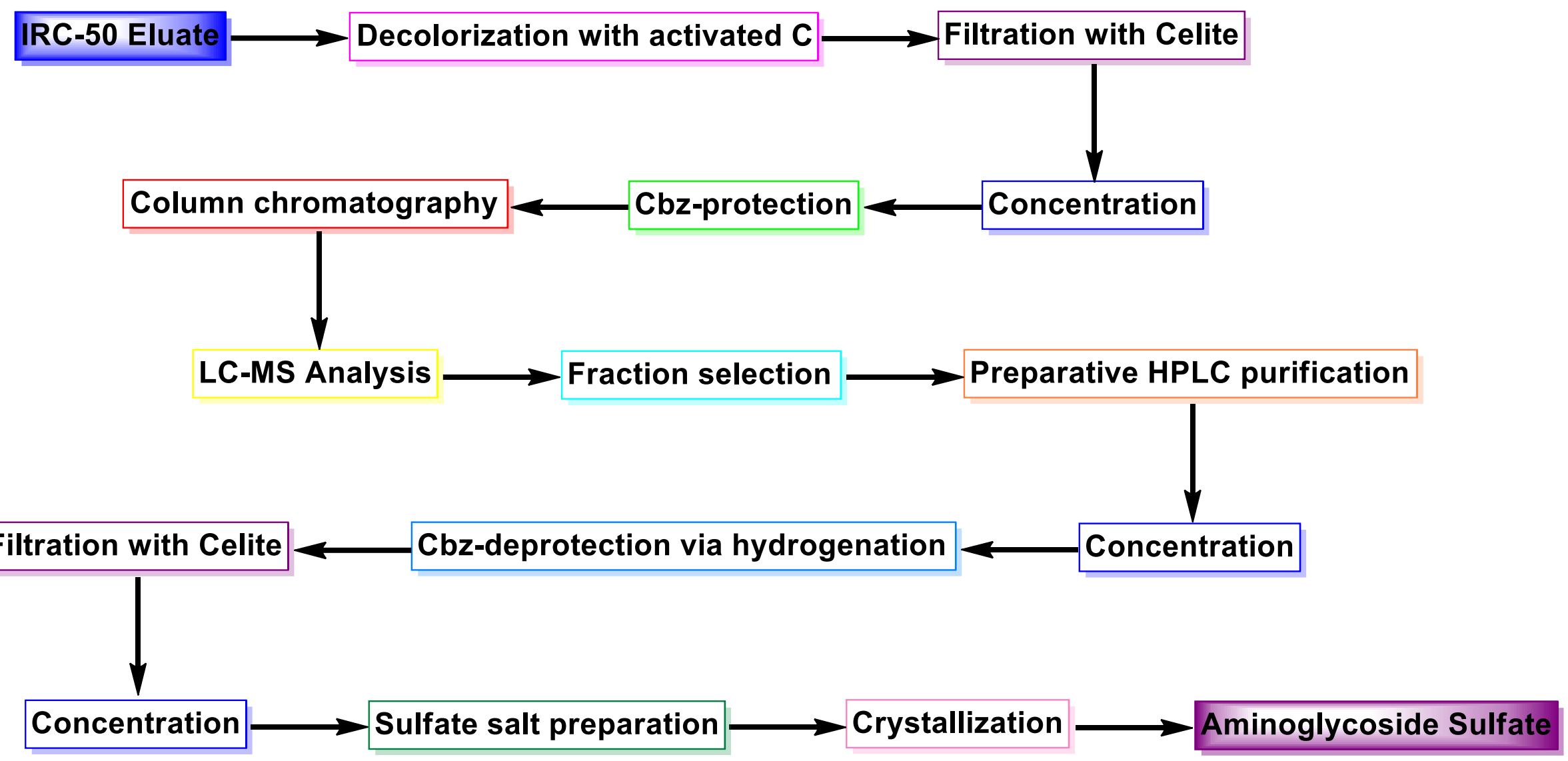

Figure S4. Isolation of GM aminoglycosides obtainable from the IRC-50 eluate

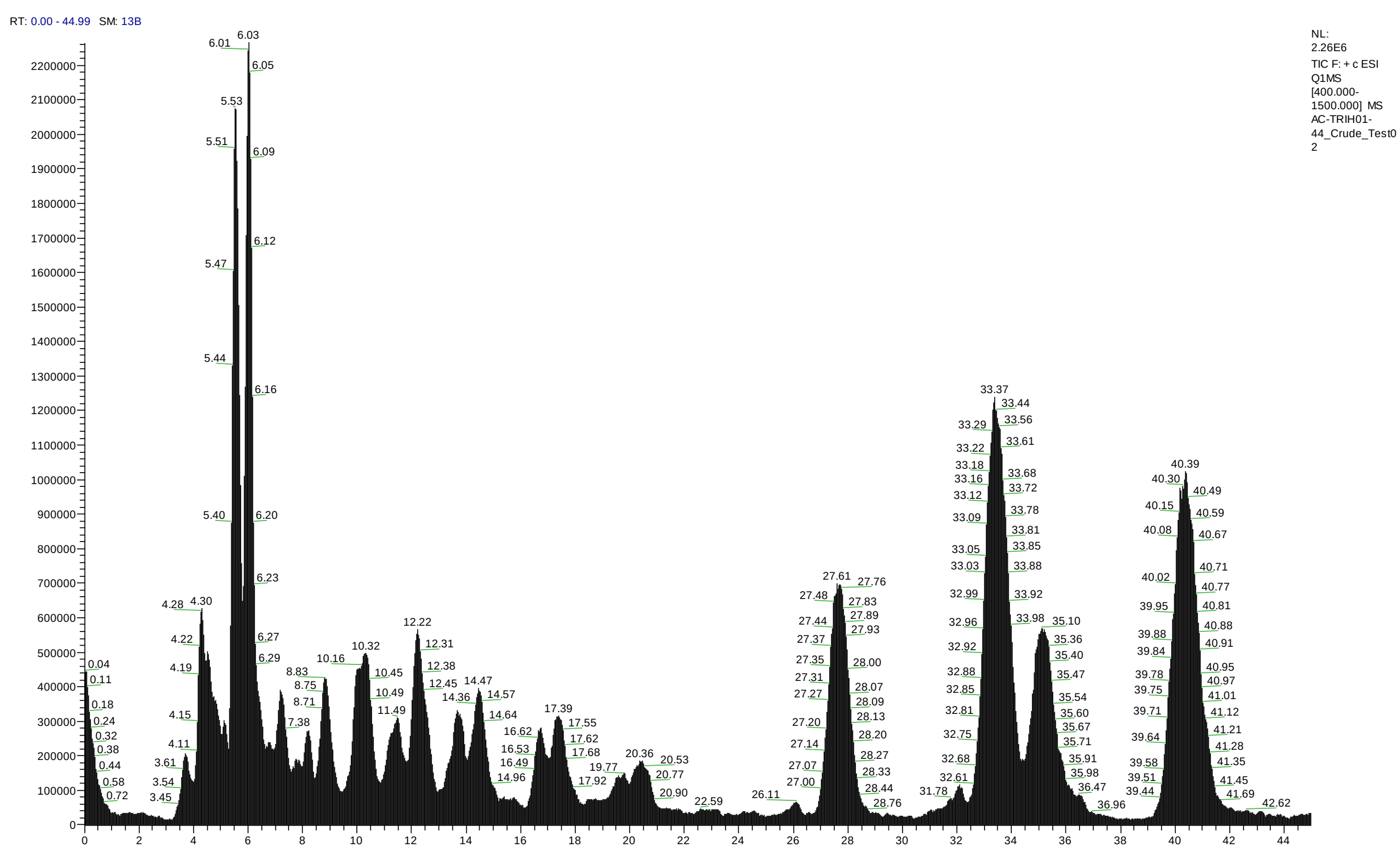

Figure S5. UV chromatogram (HPLC/MS) of crude mixture obtained *via* Cbz-protection of aminoglycosides from the IRC-50 eluate. The major components of the mixture correspond to Cbz-protected GM C1a (t_R = 27.61 min), Cbz-protected GM C2 (t_R = 33.37 min), Cbz-protected GM

C2a (t_R = 35.10 min) and Cbz-protected GM C1 (t_R = 40.39 min). UV signals stretching from t_R = 3,61 min to 20,53 min belong to a variety of other Cbz-protected GM congeners and their biosynthetic precursors.

Isolation of Cbz-protected GM *B1 and Cbz-protected* GM *X2 on the preparative HPLC*

The HPLC separation of Cbz-protected GM congeners was accomplished on Knauer preparative HPLC, with Smartline 2500 UV detector and 2 channel preparative pump 1800 utilizing VP150/32 Nucleodur C18 HTech 5 μm preparative column from Macherey-Nagel. The preferred separation method included an isocratic elution. The mobile phase was composed of acetonitrile/water (60/40 v/v) with 1‰ formic acid and the method length adjusted to 90 minutes. Loading capacity was between 300-400 mg of Cbz-protected GM per injection. Combined fractions from the column chromatography (enriched with Cbz-protected GM B, Cbz-protected GM B1 and Cbz-protected GM X2; fractions 41-50) were concentrated to provide a pale yellow crispy solid which was further analyzed by HPLC/MS (Figure S6). From the UV chromatogram (HPLC/MS) and MS analysis, we detected the presence of Cbz-protected GM B/Cbz-protected GM B1 (t_R = 6,65 min.) which eluted as a mixture and Cbz-protected GM X2 (t_R = 7,63 min.). The crispy residue (4,00 g) was dissolved in acetonitrile (20 mL, Concentration = 200 mg/mL) using an ultrasonic bath. The slightly turbid mixture was centrifuged for 10 minutes at 4500 rpm. The collected supernatant (2 mL HPLC loop) was utilized as an injection on the preparative HPLC. The fractions were collected in the 15 mL centrifuge tubes (Falcon tubes) and analyzed with an analytical HPLC/MS instrument. Chromatograms of HPLC purified Cbz-protected GM B, Cbz-protected GM B1 and Cbz-protected GM X2 are displayed in Figures S7, S8 and S9, respectively.

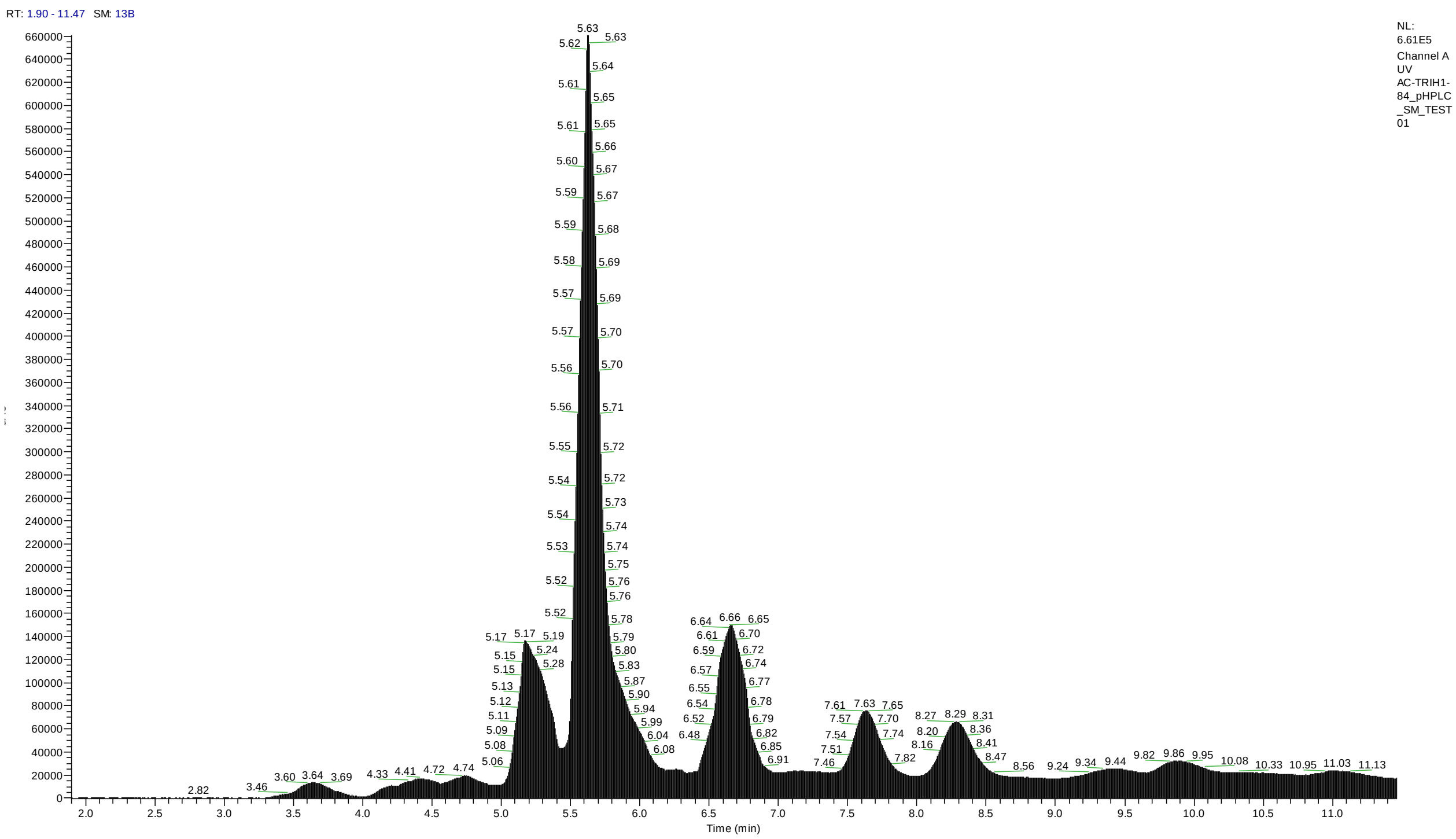

Figure S6. UV chromatogram (HPLC/MS) of combined fractions from a column chromatography (fractions 41-50). The relevant components of the mixture correspond to Cbz-protected Paromamine (t_R = 5,63 min), Cbz-protected GM B, Cbz-protected GM B1 (t_R = 6,65 min), and Cbz-protected GM X2 (t_R = 7,63 min).

a

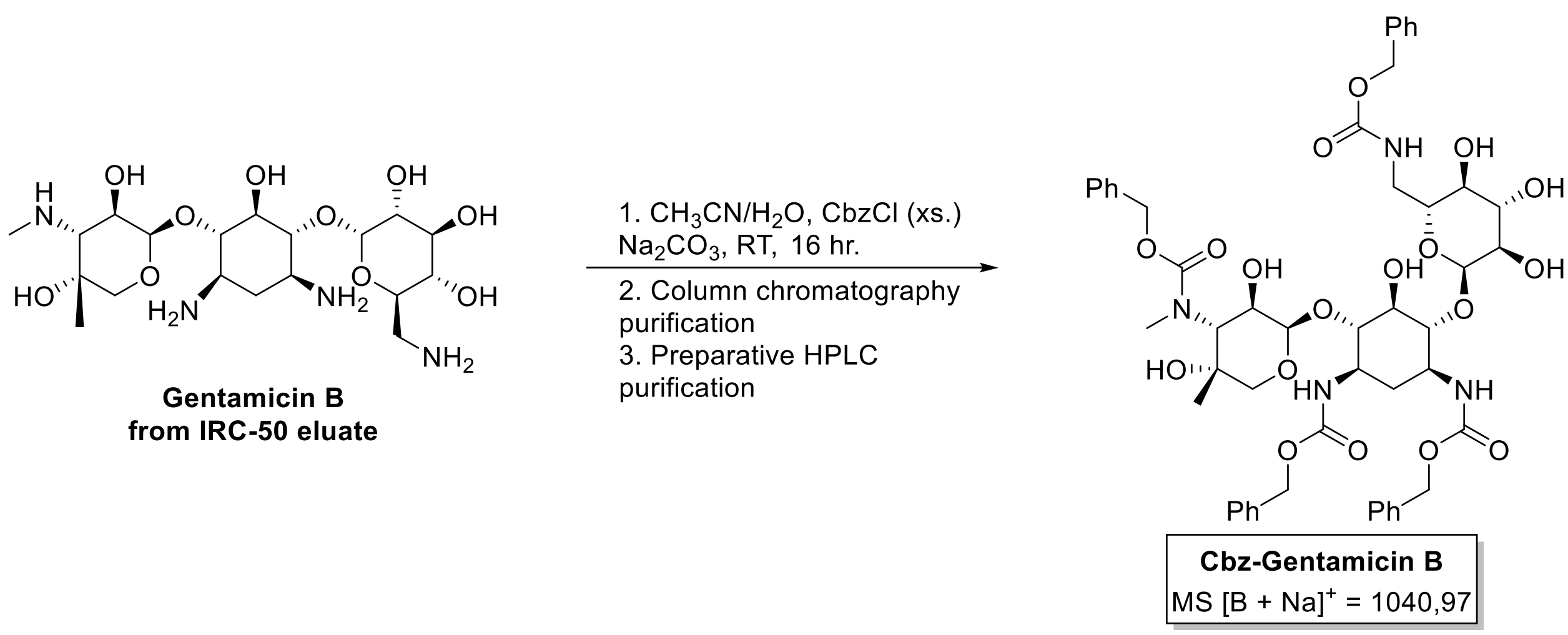

b

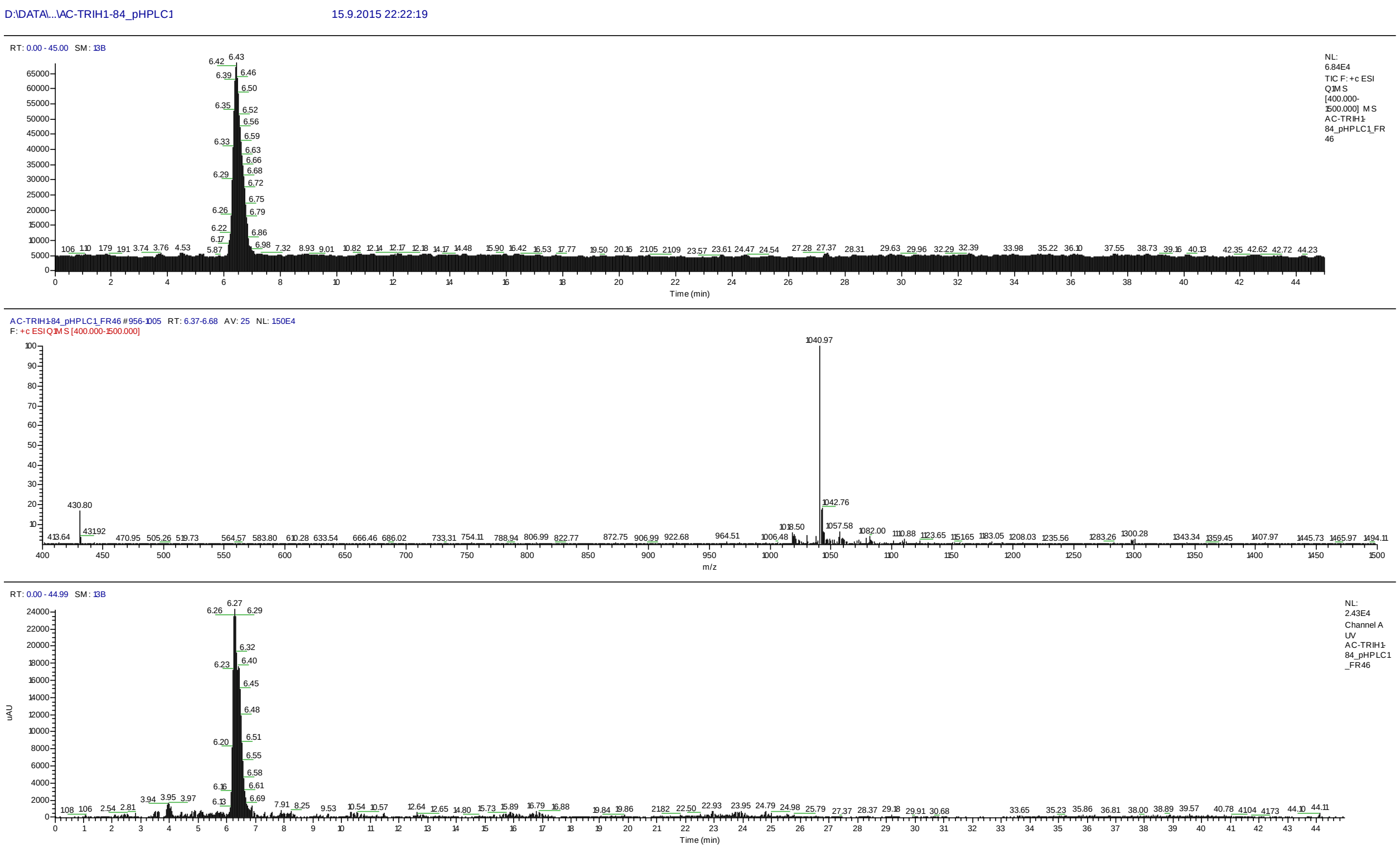

Figure S7. (a) Reaction scheme describing a derivatization protocol of GM B from IRC-50 eluate with benzyl chloroformate (Cbz-Cl). (b) Representative HPLC/MS chromatogram for Cbz-protected GM B purified with preparative HPLC (t_R = 6.27 min., MS $[B + Na]^+$ = 1040.97).

a

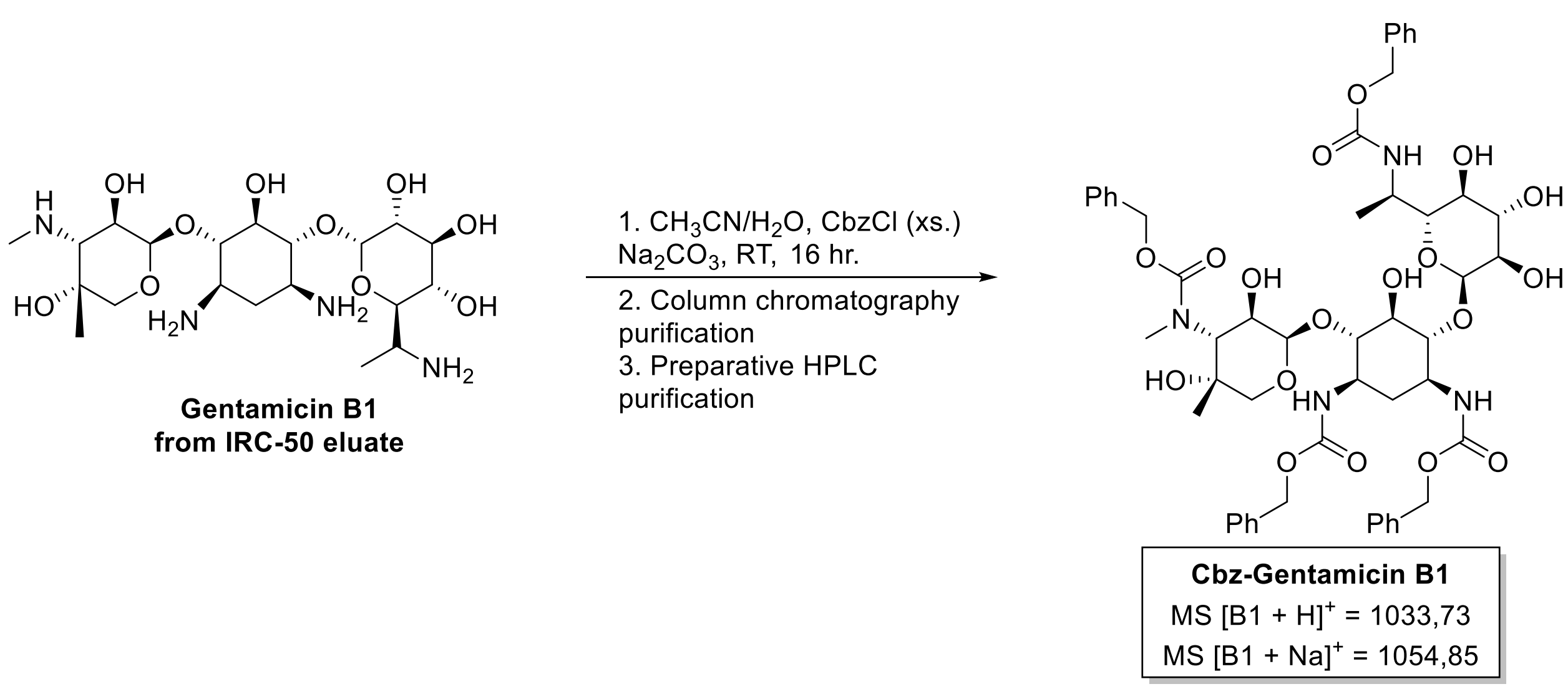

b

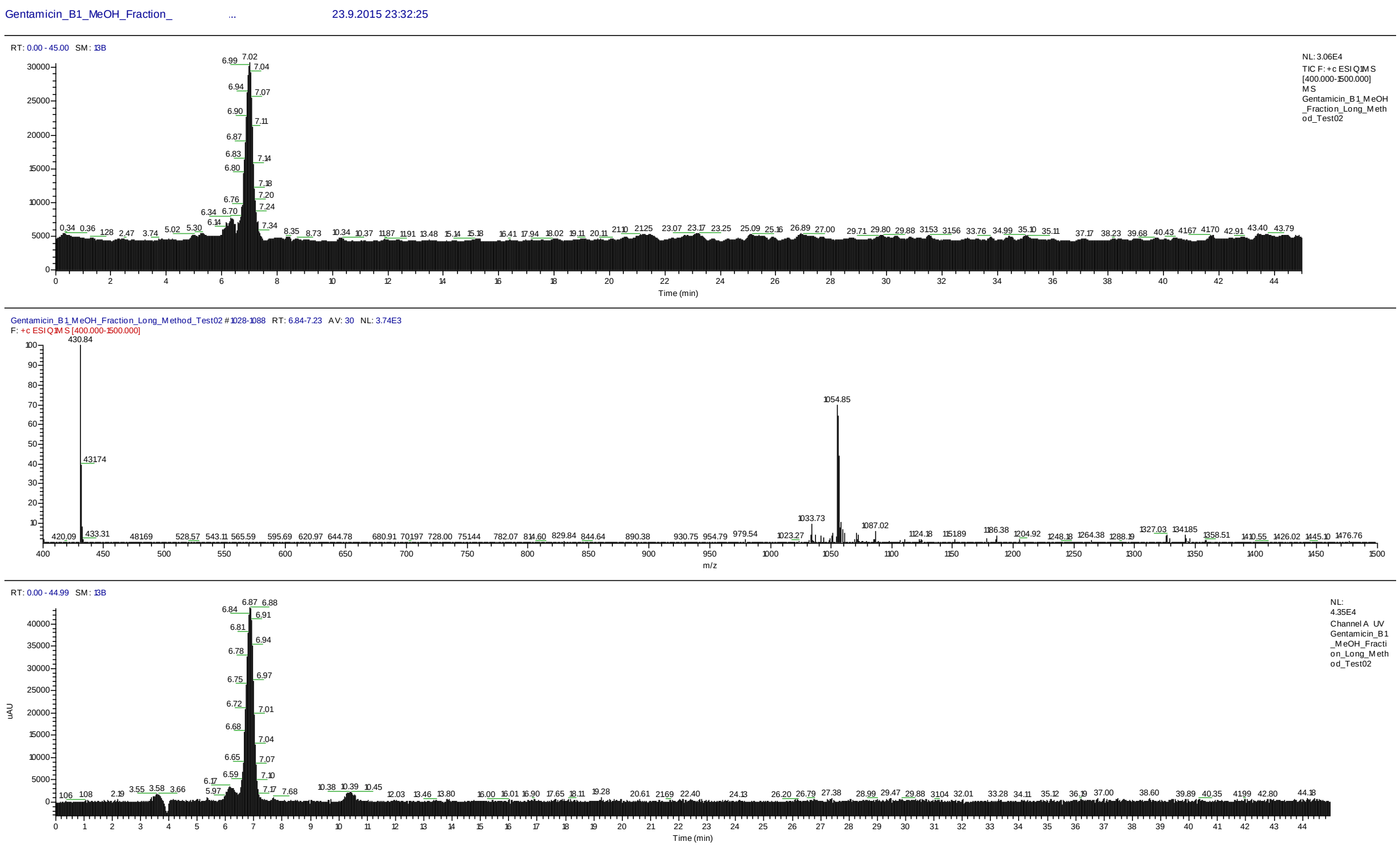

Figure S8. (a) Reaction scheme describing a derivatization protocol of GM B1 from IRC-50 eluate with benzyl chloroformate (Cbz-Cl). (b) Representative HPLC/MS chromatogram for Cbz-protected GM B1 purified with preparative HPLC (t_R = 6.88 min., MS $[B1 + H]^+$ = 1033.73; MS $[B1 + Na]^+$ = 1054.85).

a

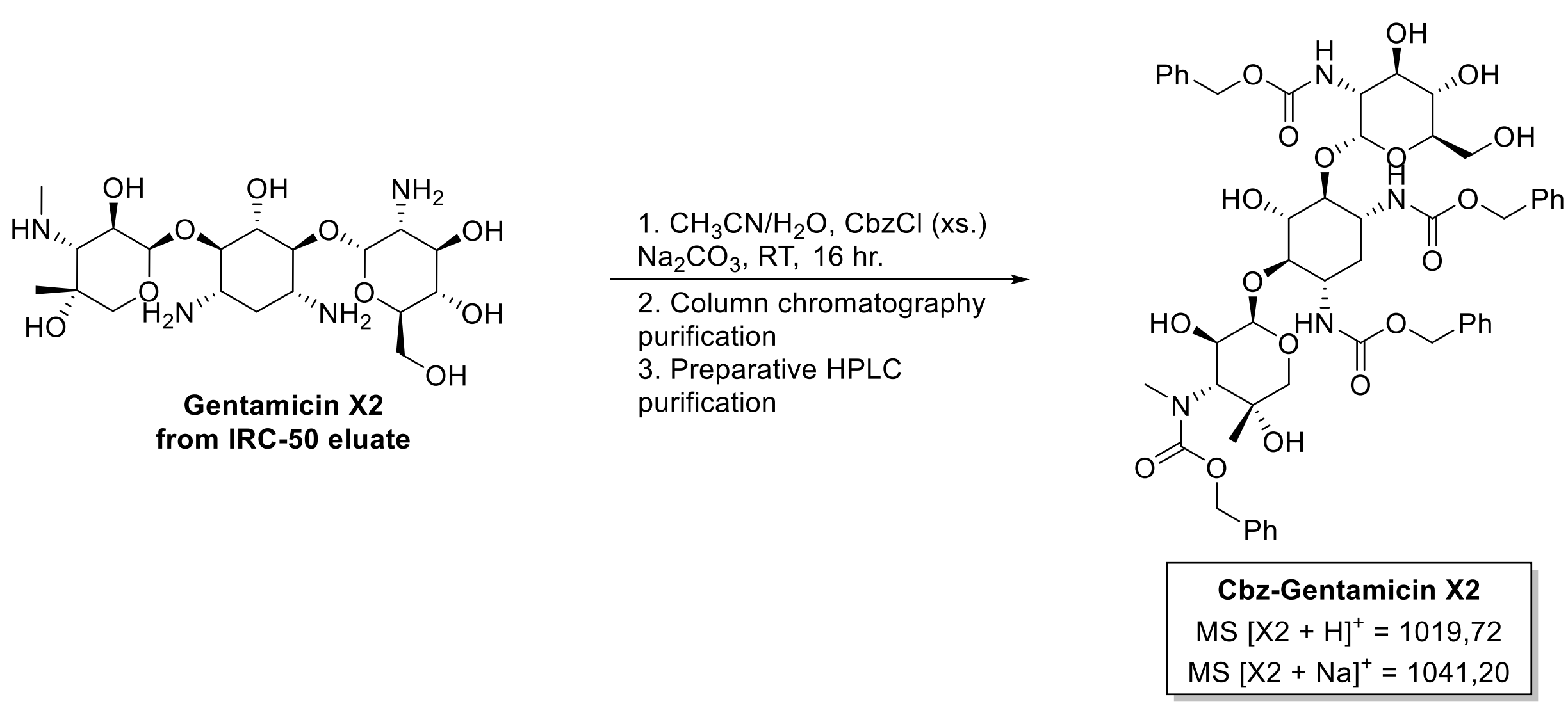

b

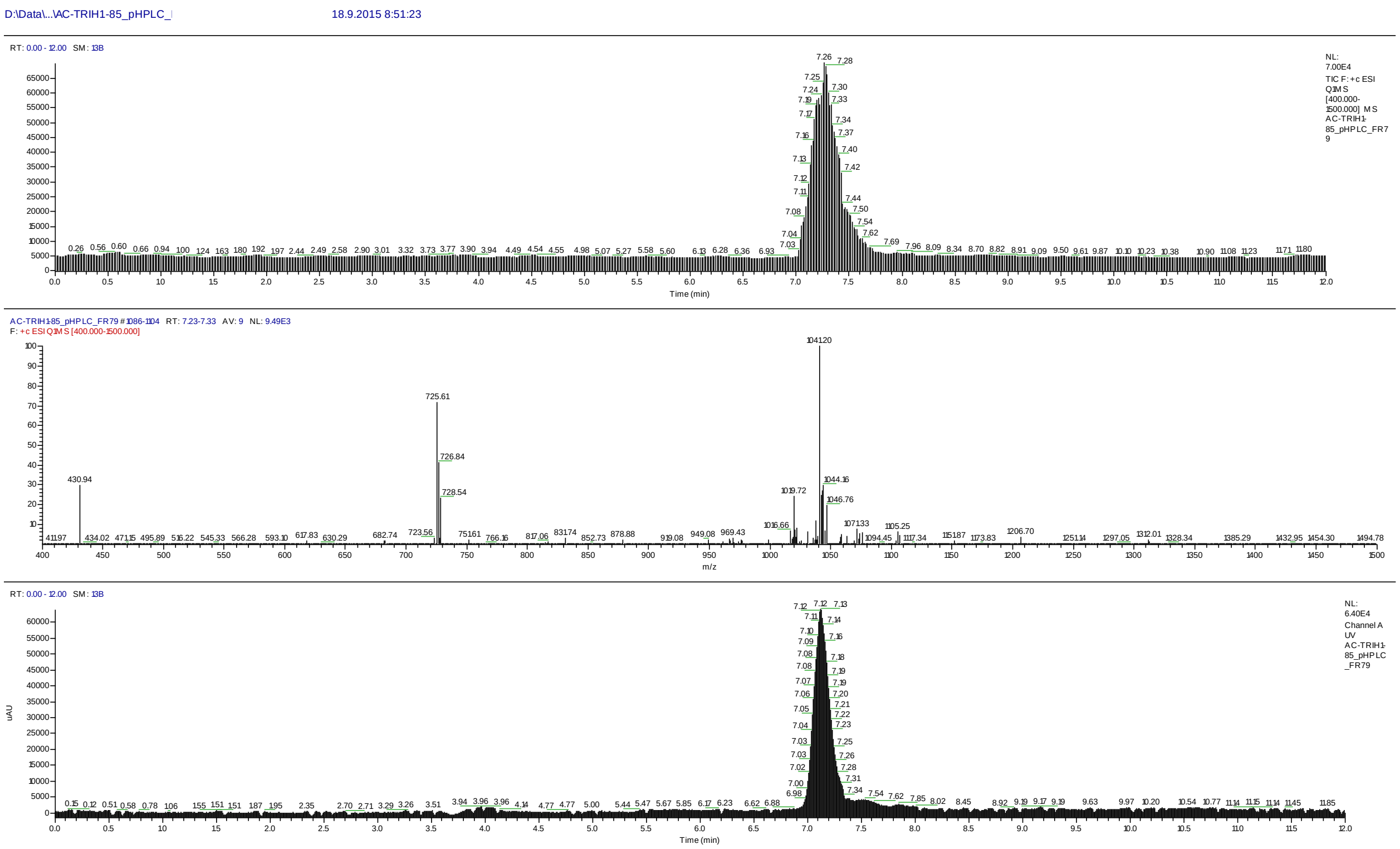

Figure S9. (a) Reaction scheme describing a derivatization protocol of GM X2 from IRC-50 eluate with benzyl chloroformate (Cbz-Cl). (b) Representative HPLC/MS chromatogram for Cbz-protected GM X2 purified with preparative HPLC (t_R = 7.13 min., MS $[X2 + H]^+$ = 1019.72; MS $[X2 + Na]^+$ = 1041.20).

Pd-Catalyzed hydrogenation of pure Cbz-protected GM B congener

The Pd-catalyzed hydrogenation of Cbz-protected GM B congener was performed at ambient temperature (25 °C) and at 1 bar pressure of H_2 gas (Figure S10a). The vacuum-dried Cbz-protected GM B (71,3 mg, 0,070 mmol, 1.0 equiv.) was dissolved in methanol (5,0 mL). Palladium on carbon (10%, Pd/C, 213 mg/mmol of substrate, 14,9 mg) was added in a single portion, the flask evacuated and filled with hydrogen 3 times. The black suspension was stirred at ambient temperature, under 1 bar of H_2 pressure (balloon) for 16 hours. The suspension was filtered over celite filter aid, washed with methanol and concentrated. The oily residue (GM B free base, 33,8 mg, 0,070 mmol,

1.0 equiv.) was dissolved in distilled water (10,0 mL, pH = 9,34) and treated with 1.0 M H_2SO_4 (0,140 mL, 0,140 mmol, 2.0 equiv., pH = 3.5 - 4.0) to provide a disulfate salt, a more stable form of GM. Water was evaporated and the residue dried in high vacuum to provide GM B x $2H_2SO_4$ (46,8 mg, 0,069 mmol, 99 % yield) in the form of crystalline powder. Pure GM B disulfate was analyzed by LC/MS and its mass confirmed by MS spectrometry (Figure S10b).

a

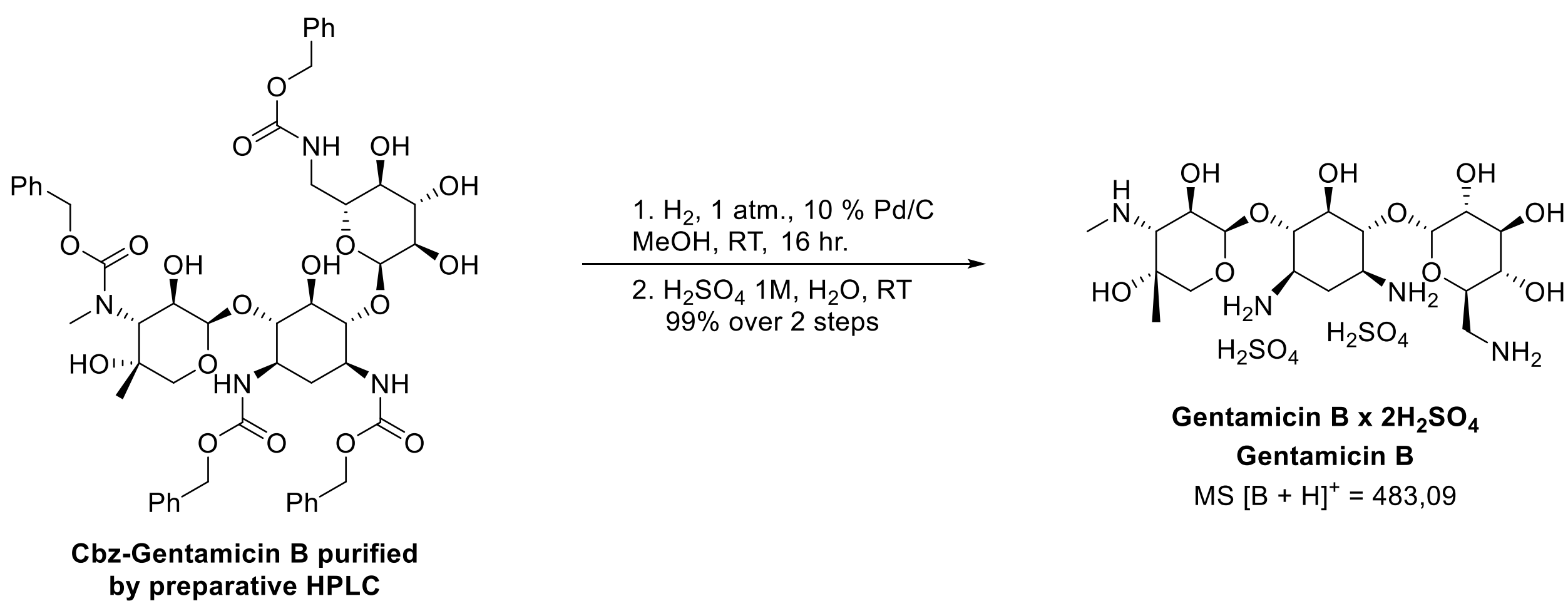

b

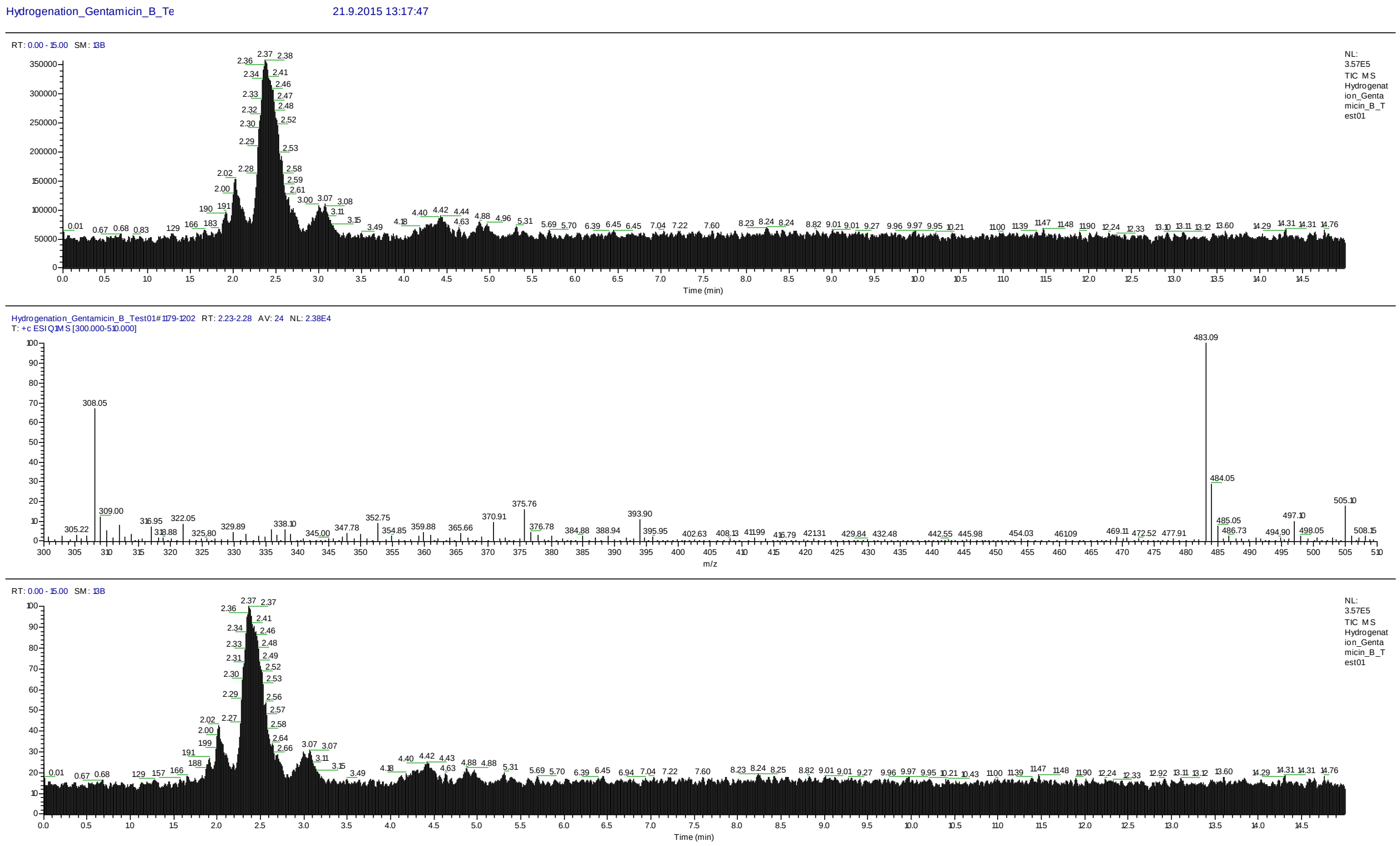

Figure S10. (a) Reaction scheme describing Cbz group deprotection from Cbz-protected GM B by Pd-catalyzed hydrogenation and subsequent disulfate formation. (b) Representative HPLC/MS chromatogram of GM B disulfate (t_R = 2.37 min., MS $[B + H]^+$ = 483.09).

Pd-Catalyzed hydrogenation of pure Cbz-protected GM B1 congener

The Pd-catalyzed hydrogenation of Cbz-protected GM B1 congener was performed at ambient temperature (25 °C) and at 1 bar pressure of H_2 gas (Figure S11a). The vacuum dried Cbz-protected GM B1 (71,3 mg, 0,069 mmol, 1.0 equiv.) was dissolved in methanol (5,0 mL). Palladium on carbon (10%, Pd/C, 213 mg/mmol of substrate, 14,7 mg) was added in a single portion, the flask evacuated and filled with hydrogen 3 times. The black suspension was stirred at ambient temperature, under 1 bar of H_2 pressure (balloon) for 16 hours. The suspension was filtered over

celite filter aid, washed with methanol and concentrated. The oily residue (GM B1 free base, 34,3 mg, 0,069 mmol, 1.0 equiv.) was dissolved in distilled water (10,0 mL, pH = 9,64) and treated with 1.0 M H_2SO_4 (0,138 mL, 0,138 mmol, 2.0 equiv., pH = 3.5 - 4.0) to provide a disulfate salt, a more stable form of GM. Water was evaporated and the residue dried in high vacuum to provide GM B1 x $2H_2SO_4$ (47 mg, 0,068 mmol, 99 % yield) in the form of crystalline powder. Pure GM B1 disulfate was analyzed by LC/MS and its mass confirmed by MS spectrometry (Figure S11b).

a

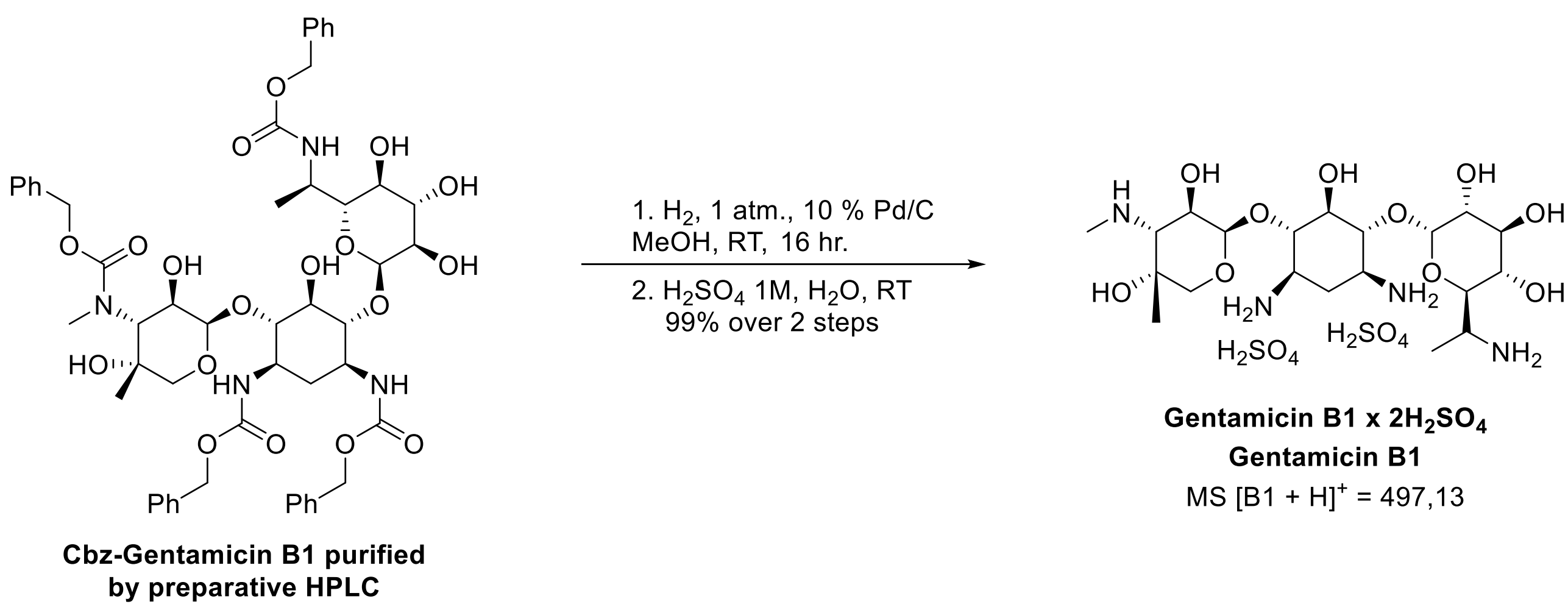

b

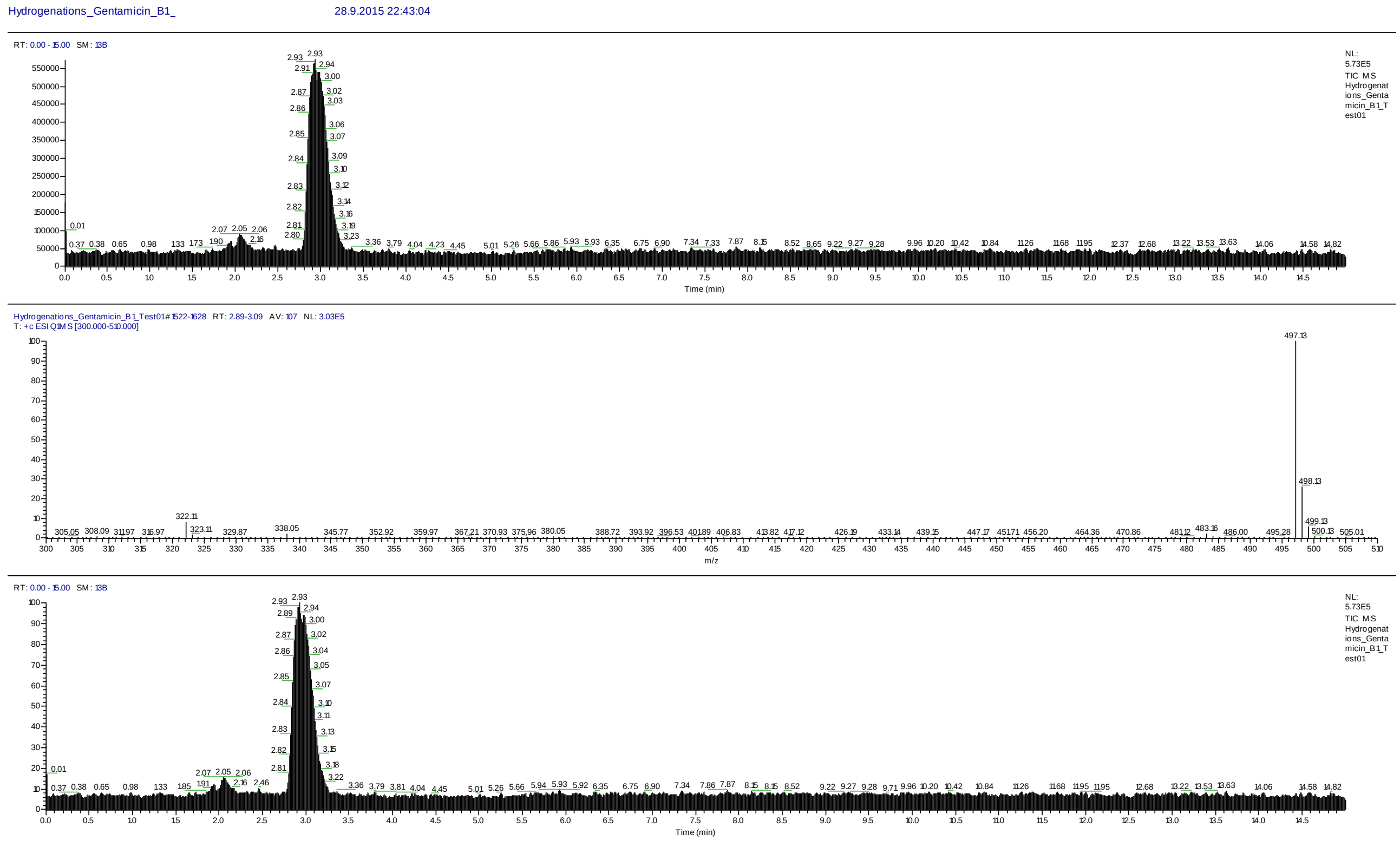

Figure S11. (a) Reaction scheme describing Cbz group deprotection from Cbz-protected GM B1 by Pd-catalyzed hydrogenation and subsequent disulfate formation. (b) Representative HPLC/MS chromatogram of GM B1 disulfate (t_R = 2.93 min., MS $[B1 + H]^+$ = 497.13).

Pd-Catalyzed hydrogenation of pure Cbz-protected GM X2 congener

The Pd-catalyzed hydrogenation of Cbz-protected GM X2 congener was performed at ambient temperature (25 °C) and at 1 bar pressure of H_2 gas (Figure S12a). The vacuum dried Cbz-protected GM X2 (122,3 mg, 0,12 mmol, 1.0 equiv.) was dissolved in methanol (5,0 mL). Palladium on carbon (10%, Pd/C, 213 mg/mmol of substrate, 25,6 mg) was added in a single portion, the flask evacuated and filled with hydrogen 3 times. The black suspension was stirred at ambient temperature, under 1 bar of H_2 pressure (balloon) for 16 hours. The suspension was filtered over

celite filter aid, washed with methanol and concentrated. The oily residue (GM X2 free base, 57,9 mg, 0,12 mmol, 1.0 equiv.) was dissolved in distilled water (10,0 mL, pH = 9,84) and treated with 1.0 M H_2SO_4 (0,24 mL, 0,24 mmol, 2.0 equiv., pH = 3.5 - 4.0) to provide a disulfate salt, a more stable form of GM. Water was evaporated and the residue dried in high vacuum to provide GM X2 x $2H_2SO_4$ (81 mg, 0,12 mmol, 99 % yield) in the form of crystalline powder. Pure GM X2 disulfate congener was analyzed by LC/MS and its structure confirmed by MS spectrometry (Figure S12b).

a

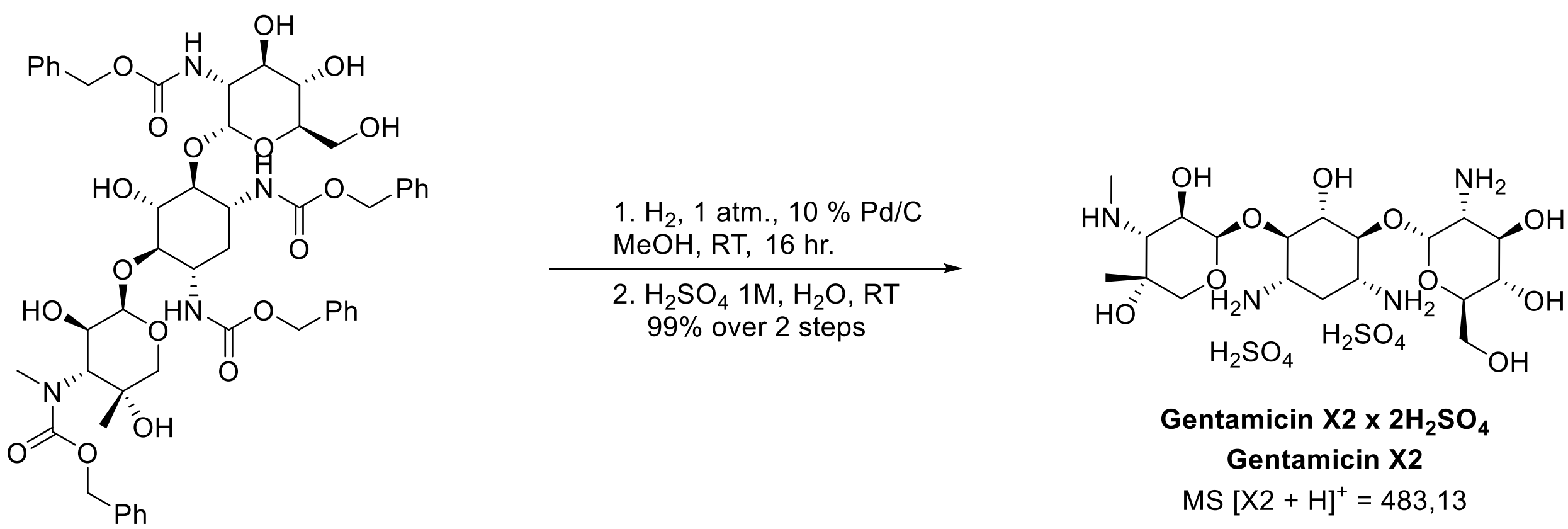

Cbz-Gentamicin X2 purified by preparative HPLC

Gentamicin X2 x $2H_2SO_4$
Gentamicin X2
MS $[X2 + H]^+$ = 483,13

b

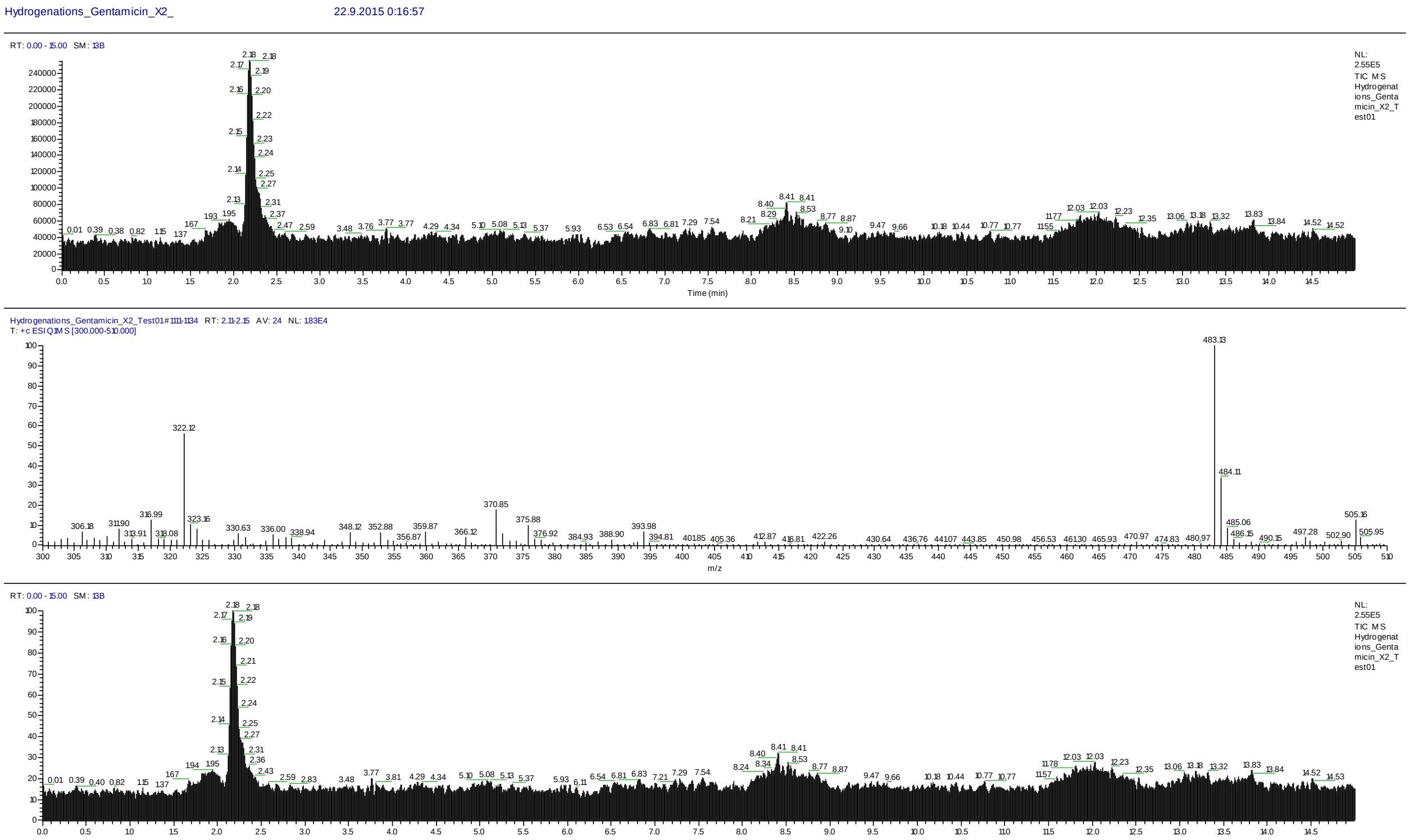

Figure S12. (a) Reaction scheme describing Cbz group deprotection from Cbz-protected GM X2 by Pd-catalyzed hydrogenation and subsequent disulfate formation. (b) Representative HPLC/MS chromatogram of GM X2 disulfate (t_R = 2.18 min., MS $[X2 + H]^+$ = 483.13).

Analytical method used for the assessment of the GM B, B1 and X2 congeners

The method required the use of a Thermo Acclaim RSLC PA2 100x2.1 mm HPLC column with a particle size of 2.2 µm. The mobile phase consisted of: 0.025% HFBA (hexafluoro butyric acid), 5% acetonitrile and 95% water (Component A), and 0.3% TFA (trifluoroacetic acid), 5% acetonitrile and 95% water (Component B). The mobile phase was pre-mixed in a ratio 1:1 and HPLC was operated at a mobile phase flow of 200 µl/min and column oven temperature of 60°C. The ESI probe parameters are described in Table S5.

Parameter:	Value:
Spray voltage:	3500 V
Vaporiser temperature:	350 °C
Sheath gas pressure:	20
Ion sweep gas pressure:	0
Aux gass pressure:	10
Capillary temperature:	150 °C
Tube lens offset:	106
Skimmer offset:	0

Table S5. hESI$^+$ probe parameters for LC-MS method

Reconfirmation of the structures of the GM B1 and X2 congeners by high-resolution mass spectrometry (HRMS)

In order to enable the efficient separation of GM B, GM B1 and GM X2, we initially generated Cbz-protected GM congeners. This step did not only ensure sufficient separation of these three congeners, but also provided a clear distinction of GM B from the GM X2 congener, which otherwise in unprotected form do have almost identical MS. The expected mass of protected Cbz-GM B and Cbz-GM X2 can be clearly distinguished with MS $[B + Na]^+$ of 1054,85 and MS $[X2 + Na]^+$ of 1041,20 (see above).

Considering that in our experiments GM B1 and GM X2 displayed a very high toxicity, we completed HRMS with these two GM congeners:

GM B1 congener

HRMS (ESI$^+$) analysis confirmed the molecular composition of GM B1. The spectrum showed a prominent ion at m/z 497.2822, corresponding to the protonated molecular ion $[M + H]^+$ of the molecular formula $C_{20}H_{41}O_{10}N_4$. This value is in excellent agreement with the calculated exact mass of 497.28172, giving a mass error of 0.96 ppm. The isotopic pattern showed high consistency

with the proposed elemental composition, with a combined score of 97.92% and full isotopic pattern coverage.

GM X2 congener

HRMS (ESI^+) analysis of GM X2 revealed a major ion at m/z 483.2661, assigned to the protonated molecular ion $[M + H]^+$, corresponding to the molecular formula $C_{19}H_{39}O_{10}N_4$. The experimentally measured mass closely matched the calculated exact mass of 483.26607, with a mass deviation of only 0.09 ppm. The observed isotopic distribution was fully consistent with the theoretical pattern, yielding a combined score of 99.04% and confirming the proposed molecular formula.

References

Clinical and Laboratory Standards Institute (2012). 'Methods for dilution antimicrobial susceptibility tests for bacteria that grow aerobically, 9th ed. Approved standard M07-A9. '. *Clinical and Laboratory Standards Institute, Wayne, PA*.

Ishikawa, M., García-Mateo, N., Čusak, A., López-Hernández, I., Fernández-Martínez, M., Müller, M., et al. (2019). 'Lower ototoxicity and absence of hidden hearing loss point to gentamicin C1a and apramycin as promising antibiotics for clinical use'. *Scientific reports*. Online. Vol. 9, no. 1. Available at: https://pubmed.ncbi.nlm.nih.gov/30787404/ [Accessed 19th October 2025].

Magiorakos, A. P., Srinivasan, A., Carey, R. B., Carmeli, Y., Falagas, M. E., Giske, C. G., et al. (2012). 'Multidrug-resistant, extensively drug-resistant and pandrug-resistant bacteria: An international expert proposal for interim standard definitions for acquired resistance'. *Clinical Microbiology and Infection*. Online. Vol. 18, no. 3, pp. 268–281. Available at: https://pubmed.ncbi.nlm.nih.gov/21793988/ [Accessed 12th October 2025].
